# Language Experience Impacts Brain Activation for Spoken and Signed Language in Infancy: Insights From Unimodal and Bimodal Bilinguals

**DOI:** 10.1162/nol_a_00001

**Published:** 2020-04-06

**Authors:** Evelyne Mercure, Samuel Evans, Laura Pirazzoli, Laura Goldberg, Harriet Bowden-Howl, Kimberley Coulson-Thaker, Indie Beedie, Sarah Lloyd-Fox, Mark H. Johnson, Mairéad MacSweeney

**Affiliations:** 1 Goldsmiths, University of London, London, UK; 2 University College London, London, UK; 3 Birkbeck - University of London, London, UK; 4 University of Westminster, London, UK; 5 Boston Children’s Hospital, Boston, Massachusetts, US; 6 University of Plymouth, Plymouth, Devon, UK; 7 University of Hertfordshire, Hatfield, Hertforshire, UK; 8 University of Cambridge, Cambridge, Cambridgeshire, UK

**Keywords:** deaf, fNIRS, infants, sign language, speech, infant-directed language

## Abstract

Recent neuroimaging studies suggest that monolingual infants activate a left-lateralized frontotemporal brain network in response to spoken language, which is similar to the network involved in processing spoken and signed language in adulthood. However, it is unclear how brain activation to language is influenced by early experience in infancy. To address this question, we present functional near-infrared spectroscopy (fNIRS) data from 60 hearing infants (4 to 8 months of age): 19 monolingual infants exposed to English, 20 unimodal bilingual infants exposed to two spoken languages, and 21 bimodal bilingual infants exposed to English and British Sign Language (BSL). Across all infants, spoken language elicited activation in a bilateral brain network including the inferior frontal and posterior temporal areas, whereas sign language elicited activation in the right temporoparietal area. A significant difference in brain lateralization was observed between groups. Activation in the posterior temporal region was not lateralized in monolinguals and bimodal bilinguals, but right lateralized in response to both language modalities in unimodal bilinguals. This suggests that the experience of two spoken languages influences brain activation for sign language when experienced for the first time. Multivariate pattern analyses (MVPAs) could classify distributed patterns of activation within the left hemisphere for spoken and signed language in monolinguals (proportion correct = 0.68; *p* = 0.039) but not in unimodal or bimodal bilinguals. These results suggest that bilingual experience in infancy influences brain activation for language and that unimodal bilingual experience has greater impact on early brain lateralization than bimodal bilingual experience.

## Introduction

Areas of the frontal and temporal cortex are crucial to language processing in adulthood. These regions are already activated in response to spoken language in the first few days or weeks of life (Altvater-Mackensen & Grossmann, 2016; Dehaene-Lambertz, Dehaene, & Hertz-Pannier, 2002; Dehaene-Lambertz et al., 2006, 2010; May, Gervain, Carreiras, & Werker, 2018; Minagawa-Kawai et al., 2010; Pena et al., 2003; Perani et al., 2011; Sato et al., 2012; Shultz, Vouloumanos, Bennett, & Pelphrey, 2014; Vannasing et al., 2016). As in adults, brain responses to speech in infants are often found to be greater in amplitude in the left than in the right hemisphere (Altvater-Mackensen & Grossmann, 2016; Dehaene-Lambertz et al., 2002, 2010; Minagawa-Kawai et al., 2010; Pena et al., 2003; Shultz et al., 2014; Vannasing et al., 2016), but this left lateralization is not always observed in infants (Dehaene-Lambertz et al., 2006; May, Byers-Heinlein, Gervain, & Werker, 2011; May et al., 2018; Perani et al., 2011).

The similarities between the adult and infant language networks suggest an early neural specialization for language processing in human development. However, the presence of this pattern from birth does not necessarily imply that it is established in the absence of experience. A fetus can hear from the 24th to 25th gestational week (Birnholz & Benacerraf, 1983). The preference of neonates for their mother’s voice and the language heard in utero indicate that fetuses use the sounds and vibrations of their mother’s voice to begin learning the foundations of voice and language processing prenatally (DeCasper & Fifer, 1980; Moon, Cooper, & Fifer, 1993). Even in preterm newborns (born at 28 to 32 weeks of gestation), who have very limited prenatal experience of language, some aspects of the neural circuits for language appear to be already in place. Indeed the response to syllables in the posterior temporal cortex of these preterm infants is faster and more sustained in the left than the right hemisphere (Mahmoudzadeh et al., 2013).

Despite this early neural specialization for language, there is also a clear role of experience in shaping the neural substrate of language. In newborns and in older infants, brain activation for the familiar language has been shown to be larger in amplitude (Fava, Hull, & Bortfeld, 2014; May et al., 2011; Minagawa-Kawai et al., 2010; Sato et al., 2012) and more left lateralized (Sato et al., 2012; Vannasing et al., 2016) than for an unfamiliar language. Moreover, in the left temporal and temporoparietal cortex of newborns, forward speech elicits more activation than backward speech for a familiar language, whereas this difference is not observed for an unfamiliar language (May et al., 2018; Sato et al., 2012; but see also May et al., 2011).

Another way of assessing how experience shapes the neural substrate of language is by comparing infants with different language experience, such as monolinguals and bilinguals. When infants are exposed to two spoken languages from birth, they acquire two linguistic codes (two sets of sounds, two lexicons, two sets of grammatical rules) and learn to keep them apart, even though they experience a reduced amount of each of these codes compared to monolinguals (Costa & Sebastián-Gallés, 2014; Werker, 2012). Although this process is extremely complex, bilinguals usually follow the same milestones of early language development that monolinguals follow (Costa & Sebastián-Gallés, 2014; Werker, 2012). It has been suggested that native and non-native phonetic contrasts are differently represented in the brains of bilingual compared to monolingual infants (Ferjan Ramírez, Ramírez, Clarke, Taulu, & Kuhl, 2017; Garcia-Sierra et al., 2011; Petitto et al., 2012). To our knowledge, there are no published studies comparing the neural responses to familiar and unfamiliar languages in bilingual and monolingual babies above the phonetic level.

Hearing infants with deaf mothers who use a sign language also grow up to be bilingual. They offer a unique window into experience-dependent plasticity. If a deaf mother uses a sign language (such as British Sign Language [BSL]), as her preferred mode of communication, the speech and language experience of her hearing infant is likely to be very different from that of hearing infants of hearing mothers. Prenatally, if the mother is using mainly sign language in her daily interactions, the fetus is likely to have a reduction in language experience, given the reduced exposure to maternal voice in utero. After birth, hearing infants with deaf mothers experience a sign language, such as BSL, and a spoken language, such as English, which can be used by the mother, as well as by hearing relatives and the rest of the hearing community. For this reason, these infants can be referred to as “bimodal bilinguals,” as opposed to “unimodal bilinguals,” who are exposed to two spoken languages. The language produced by a deaf mother to and around her infant is likely to include less audiovisual spoken language than that of a hearing mother. Many deaf signers may use speech to communicate with hearing people, but the extent to which they actually “voice” their speech and produce sound, as opposed to silently mouth, is extremely variable (Bishop & Hicks, 2005). When addressing her infant, a deaf mother may use signed and/or spoken language, but spoken utterances by deaf mothers tend to be reduced in length and frequency compared to that of hearing mothers (Woll & Kyle, 1989).

Studies that have addressed spoken language development in bimodal bilingual children are usually based on a single child or a few children, and they often report inconsistent results. Early vocabulary development in bimodal bilinguals has been reported to be similar to that of monolinguals (Brackenbury, Ryan, & Messenheimer, 2006; Capirci, Iverson, Montanari, & Voltera, 2002; Griffith, 1985), better than monolinguals (Daniels, 1993), or poorer than monolinguals (Murphy & Slorach, 1983; Schiff-Meyers, 1993). Poorer performance in bimodal bilingual children compared to monolingual norms has also been reported on assessments of phonology, comprehension, and/or grammar (Hofmann & Chilla, 2015; Johnson, Watkins, & Rice, 1992; Murphy & Slorach, 1983; Schiff & Ventry, 1976; Schiff-Meyers, 1993). However, because bimodal bilinguals grow up learning two languages, a more appropriate contrast is with unimodal bilinguals than with monolinguals. Such comparison suggests that bimodal bilinguals may achieve the early linguistic milestones in spoken and in signed language at the same time as children learning two spoken languages (Hofmann & Chilla, 2015; Petitto et al., 2001).

It is unclear how bimodal bilingual experience affects brain activation for spoken language in infancy. Moreover, the neural representation for sign language has never been studied in infancy. Adult neuroimaging studies robustly demonstrate that sign language is processed in a brain network similar to that of spoken language in deaf and hearing adults who are fluent in sign language (Capek et al., 2008; Emmorey, 2001; Hickok, Bellugi, & Klima, 1996; MacSweeney et al., 2004; MacSweeney, Capek, Campbell, & Woll, 2008; Petitto et al., 2000). This is a strong argument for the idea that classical language areas in the left perisylvian cortex are specialized for the processing of natural languages independent of their modality. However, it is unclear if this activation pattern in adulthood represents an adaptation that takes years of language experience and language learning to be established or if it can be observed from infancy.

The present study aims to clarify how the development of the neural system’s supporting language perception is influenced by the infant’s language experience. To address this we use functional near-infrared spectroscopy (or fNIRS). A technique that has been used successfully to study brain representation for language in infants (Altvater-Mackensen & Grossmann, 2016; Fava et al., 2014; May et al., 2011, 2018; Minagawa-Kawai et al., 2010; Pena et al., 2003; Sato et al., 2012; Vannasing et al., 2016), fNIRS measures hemodynamic responses elicited by neuronal activation. This technique offers a balance of spatial and temporal resolution, is relatively robust to movement artifacts, and requires less infant tolerance than does fMRI or MEG (Lloyd-Fox, Blasi, & Elwell, 2010). Using traditional univariate approaches to fNIRS data analysis (Aslin, Shukla, & Emberson, 2015; Lloyd-Fox et al., 2010), we describe patterns of neural activation in response to spoken and signed language in 60 hearing infants. Furthermore, we assess how these patterns of language activation for spoken and signed language differ in three groups of infants with different language experience: monolinguals, unimodal bilinguals, and bimodal bilinguals. Using region-of-interest (ROI) analyses, we test the hypothesis that, in response to spoken language, bimodal bilinguals show reduced amplitude of activation and reduced lateralization in frontotemporal language areas compared with monolinguals and unimodal bilinguals, due to reduced input of auditory spoken language from their mothers. On the other hand, we predict that bimodal bilinguals will show increased amplitude of activation in frontotemporal language areas and increased lateralization in response to sign language compared to monolinguals and unimodal bilinguals, who have never experienced sign language.

A second aim of the current study is to clarify how the effects of language familiarity on brain activation are influenced by an infant’s language experience. Based on the literature reviewed above, it is predicted that a familiar language, here spoken English, will be associated with increased activation compared to an unfamiliar language in all infants. Moreover, it is predicted that these familiarity effects will be reduced in both groups of bilinguals compared to monolinguals, as they are likely to have experienced a reduced amount of the familiar language, since their language exposure is split between two languages. We also predict that in bimodal bilingual infants, a familiar sign language will be associated with increased activation compared to an unfamiliar sign language. These predictions will be assessed with univariate and ROI analyses.

In addition to addressing these questions using univariate and ROI analyses, we use multivariate pattern analysis (MVPA) to compare distributed patterns of brain activation associated with spoken versus signed language, and with familiar versus unfamiliar languages. The use of multivariate analyses potentially offers greater sensitivity to experimental effects by pooling weakly discriminative information that is distributed across measurement channels (Haynes & Rees, 2006; Norman, Polyn, Detre, & Haxby, 2006). Support vector machines, in particular, are robust to the inclusion of noninformative channels, hence avoiding the arbitrary selection of channels of interest (McGettigan et al., 2012). Although fNIRS has fewer channels compared to fMRI voxels, multivariate analysis methods have been used successfully in studies in both adults and children (Bogler, Mehnert, Steinbrink, & Haynes, 2014; Gu et al., 2018; Heger, Mutter, Herff, Putze, & Schultz, 2013; Hosseini et al., 2011; Ichikawa et al., 2014; Luu & Chau, 2008; Misawa, Shimokawa, & Hirobayashi, 2014). Applying MVPA to infant fNIRS data is challenging given the low trial numbers, variability, and noise in the data. Nevertheless, Emberson, Zinszer, Raizada, and Aslin (2017) successfully used MVPA to decode visual and auditory stimuli in infant fNIRS data as well as two different types of audiovisual stimulation.

In the current study, we hypothesize that MVPA can be used to classify brain activation in response to spoken and signed language in infants who have experienced only spoken language (monolinguals and unimodal bilinguals). We also hypothesize that information in the left hemisphere will be critical to this classification of language modalities. Moreover, we hypothesize that spoken and signed language will be associated with more similar patterns of neural activity in infants who have experience of both modalities (bimodal bilinguals) compared to monolinguals and unimodal bilinguals. Therefore we predict that decoding spoken and signed language using MVPA will be more successful in monolinguals and unimodal bilinguals than in bimodal bilinguals. Finally, we hypothesize that a familiar language can be discriminated from an unfamiliar language based on distributed patterns of activation. This classification is predicted to be more successful in monolinguals than in either of the bilingual groups due to greater exposure to the familiar language in monolinguals.

## Materials and methods

### Participants

Data are presented from 60 infants between 4 and 8 months of age. An additional 34 infants participated in the study but were not included in the analyses due to equipment malfunction (n = 3), withdrawal (n = 1), channel rejection, and looking time criteria (n = 30; see Data Processing section for details). This dropout rate is similar to that for other infant fNIRS studies (Lloyd-Fox et al., 2010). Infants were from three groups with different language experience: 19 monolingual infants with hearing parents (8 girls; mean age = 6.1 months, SD = 1.0), 20 unimodal bilingual infants with hearing parents (6 girls; mean age = 6.0 months, SD = 0.9), and 21 bimodal bilingual infants with deaf parents (11 girls; mean age = 6.4 months, SD = 1.2). Age did not differ between groups (*F*(2) = 0.67; *p* = 0.518; η^2^ = 0.023) and did not differ in variance between groups (*F*(2, 57) = 1.5; *p* = 0.225; see Supplementary Figure 1A for distribution). Maternal education did not differ between groups (Pearson chi-square (6) = 6.6; *p* = 0.356; see Supplementary Figure 1B). Annual household income varied greatly between families and there was an underrepresentation of bimodal bilingual families in the highest income categories (Pearson chi-square (24) = 73.4; *p* < 0.001; see Supplementary Figure 1C). Children came from 58 different families (one family had twins and one family returned later to participate with a younger sibling). Most infants were born at term (37 to 42 weeks of gestation), except for two infants who were born slightly before term (35 to 36 weeks of gestation) for whom a corrected age was used. Infants had no severe hearing or vision problems, and no history of seizure or other serious mental or physical health issues according to their parents.

Monolingual infants were exposed to English only and both parents were hearing monolinguals. Unimodal bilinguals were frequently and regularly exposed to English and one or more additional spoken language(s) (see Supplementary Table 1 for additional languages and language combinations). All infants in this group had a hearing bilingual/multilingual mother. Most unimodal bilingual infants also had a bilingual/multilingual father (n = 15), whereas five had a monolingual father. Bimodal bilinguals were frequently and regularly exposed to BSL and English. All infants in this group had a deaf mother who used BSL as her preferred mode of communication. Nineteen bimodal bilingual infants had a severely/profoundly deaf father, one had a hearing father, and one had a single deaf mother. Infants exposed to French or French Sign Language of Belgium (LSFB) were excluded from the study because these languages were used as unfamiliar languages in the present study.

The Mullen Scales for Early Learning (Mullen, 1995) was administered to all infants to assess for any differences in development that could influence the interpretation of brain imaging results. An analysis of variance (ANOVA) with three groups on Mullen *t* scores revealed no group effect on visual reception, *F*(2) = 1.7; *p* = 0.188; η^2^ = 0.057; fine motricity, *F*(2) = 1.8; *p* = 0.183; η^2^ = 0.058, and gross motricity scales, *F*(2) = 2.2; *p* = 0.119; η^2^ = 0.072 (see Table 1). A significant effect of group was observed on the receptive language scale, *F*(2) = 3.9; *p* = 0.025; η^2^ = 0.122. Post hoc *t* tests revealed that bimodal bilinguals outperformed unimodal bilinguals (*p* = 0.013) and monolinguals (*p* = 0.028) on receptive language skills, whereas there was no difference between monolinguals and unimodal bilinguals (*p* = 0.785). The items included in this scale for this age group were mostly communicative in nature, such as interacting with their reflection in a mirror, or turning around when an experimenter called their name from behind their back. None of these items was rated by parental report. The expressive language scale was excluded because its administration disadvantaged bimodal bilinguals (see Procedure for details).

Bimodal bilinguals were recruited through social media and websites specifically aimed at the deaf community. Infants with hearing parents were contacted from the Birkbeck Babylab database of volunteers recruited from advertisements at parent-and-baby groups, parenting websites, and publications. Deaf families were geographically spread across Great Britain, while infants with hearing parents came mostly from London and its surroundings. Travel expenses were reimbursed, and a baby t-shirt and certificate of participation were offered to families. All parents provided written informed consent prior to participation, after explanations of the study in English or BSL, depending on the parents’ preferred mode of communication. The protocol was approved by the Birkbeck and UCL Research Ethics Committees and conforms to the Declaration of Helsinki.

### Stimuli

**Figure 1:**
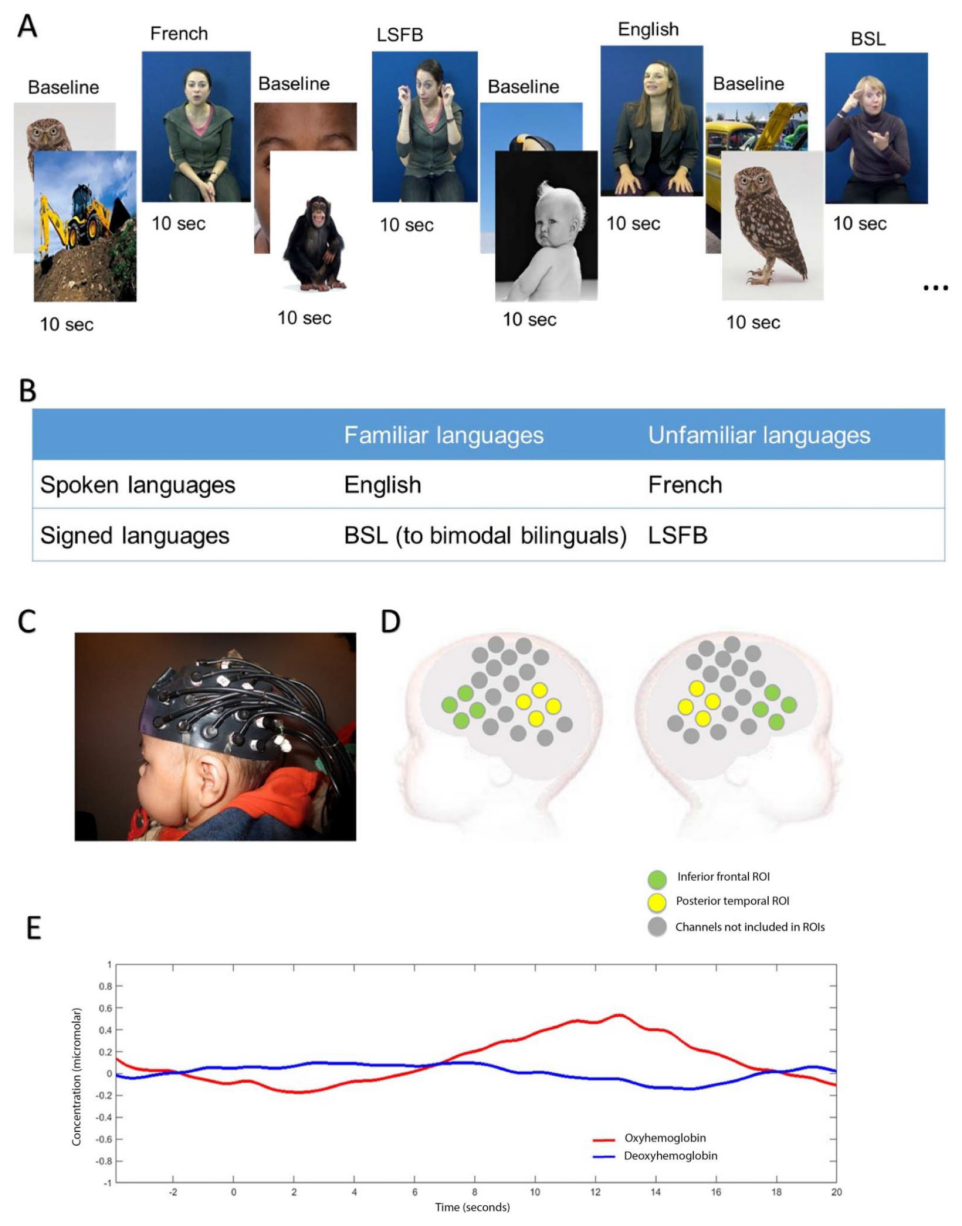
Stimuli and fNIRS measurement. A. Example of stimulus sequence and timings. B. Summary of experimental conditions and experimental design. C. Picture of infant wearing the NTS fNIRS headgear used in the current study. D. Illustration of channel location in relation to an infant’s head. Channels depicted in green are part of the inferior frontal region of interest, while channels depicted in yellow are part of the posterior temporal region of interest. E. Example of HbO_2_ and HHb grand averaged hemodynamic responses in a left posterior temporal channel in response to spoken language. Highlighted time windows represent periods of experimental stimulus and analyses.

Experimental stimuli consisted of audiovisual videos of extracts from two children’s storybooks. All infants were presented with four different experimental conditions: infant-directed English (spoken language, familiar to all infants), infant-directed French (spoken language, unfamiliar to all infants), infant-directed BSL (sign language, familiar to infants with deaf mothers), and infant-directed LSFB (sign language, unfamiliar to all infants). BSL and LSFB are parts of different families of sign languages, respectively, the British Sign Language family and the “Langue des Signes Française” family (https://glottolog.org). Four female models who were bilingual in a different combination of languages (English-French, English-BSL, French-LSFB, or BSL-LSFB) contributed four videos each (two videos per language). Videos were 9 to 12 s long (mean 10.5 s) and consisted of approximately three sentences from two children’s stories. These videos were interleaved with 10-s baseline trials in which static images of animals, babies, and modes of transportation were presented. The experiment started with the baseline condition. Experimental conditions were presented in pseudo-random order, alternating between spoken and signed language modalities (see Figure 1A).

### Experimental Design

All infants were presented with four experimental conditions, which could be merged in two language modalities: spoken languages (English + French) and sign languages (BSL + LSFB) (see Figure 1B). Two levels of analysis are presented in this article. First, modality analyses assess brain activation for spoken language (English + French), and brain activation for sign language (BSL + LSFB). Then familiarity analyses are performed within each language modality. These compare activation for a familiar spoken language (English) with activation for an unfamiliar spoken language (French) in each group of infants. Sign language familiarity analyses compare brain activation for a familiar sign language (BSL) with brain activation for an unfamiliar sign language (LSFB) within the bimodal bilingual group. Sign language familiarity analyses are not performed in monolinguals and unimodal bilinguals because both sign languages are unfamiliar to them.

### Procedure

Infants sat on their caregiver’s lap wearing a custom-built fNIRS-Centre for Brain and Cognitive Development (CBCD) headgear formed of 46 channels with a 2 cm source-detector separation (16 sources and 16 detectors), covering areas of the frontal, temporal, and temporoparietal cortex (see Figure 1C and 1D). Caregivers were instructed to prevent infants from grabbing the headgear and to refrain from interacting with their infant unless the infant became fussy or sought interaction. Infants were seated approximately 100 cm from a 117 cm plasma screen in a dimly lit and sound-attenuated room. The experiment ended once infants had viewed 20 experimental trials or if the infant became fussy or uninterested. The fNIRS data were recorded using the NTS optical topography system (Gowerlabs Ltd. L, UK) with two continuous wavelengths of source light: 770 and 850 nm at a sampling rate of 10 Hz. Infant’s behavior during the study was filmed for off-line coding of looking time.

After completion of the fNIRS study, the Mullen Scales of Early Learning was administered by a trained experimenter. During these sessions, the parent and experimenter interacted in English for hearing parents and in BSL and/or English for deaf parents. As described in the Mullen Scales of Early Learning manual, very little verbal instruction was given to infants of this age group, and when they were required, parents were asked to give these instructions to their infant and/or repeat them in the language of their choice. This method was used to avoid a potential disadvantage to bilingual infants. All sessions were filmed and the receptive language, visual reception, fine motricity, and gross motricity scales were scored off-line by two independent scorers. For items for which scores differed between scorers, an experienced third coder made the final decision. The expressive language scale was excluded because many of the items could be scored based on parental report and several deaf mothers reported not being aware of the details of their child’s babbling. Moreover, because sign language was used to communicate with deaf parents during these evaluation sessions, bimodal bilinguals were exposed to less spoken language than monolinguals and unimodal bilinguals, which could negatively affect their vocal productions during these sessions.

### Data Processing

Infant’s looking time during stimulus presentation was coded from videos by a researcher who was unfamiliar with the hypotheses. Only experimental trials in which the infant was looking at the screen for at least 60% of the trial duration were included for analyses. This criterion is similar to that for other fNIRS studies employing visual stimuli with infants (Di Lorenzo, Blasi, et al., 2019; Lloyd-Fox et al., 2013; Lloyd-Fox, Papademetriou, et al., 2014).

The fNIRS system measured the light attenuation from each source-detector pair (channel). These light attenuation measures were used to calculate changes in oxyhemoglobin (HbO_2_) and deoxyhemoglobin (HHb) chromophore concentration (μmol) and used as hemodynamic indicators of brain activity (Obrig & Villringer, 2003). Prior to conversion to concentration data, the attenuation measurements within each channel for each infant were inspected using artifact-detection thresholding algorithms (Lloyd-Fox et al., 2010 2009). Channels with poor signal readings, excess variability in the data measured with the coefficient of variation, or large baseline drifts were excluded from further analyses. Channels were excluded if the coefficient of variation of their attenuation exceeded 15% or if their normalized power was larger than 50% of the total power. This procedure aimed to exclude channels for which there was not enough light from the source reaching the corresponding detector (e.g., due to hair blocking either optode or one of the optodes being unclipped from the array), for which the noise characteristics per wavelength were significantly different, or for which channels contained strong frequency components unrelated to the experiment. The specific thresholds used were based on previous experience using the NTS Gowerlabs system (Lloyd-Fox et al., 2009), as it is recommended in infant studies to tailor thresholds to the particular study and fNIRS system employed (Di Lorenzo, Pirazzoli, et al., 2019). Infants with more than 15 rejected channels were excluded from all analyses. For each infant, the near-infrared intensity signal was lowpass filtered, using a cutoff frequency of 1.7 Hz to account for heart rate and certain types of instrumentation noise. This cut-off frequency is in line with recent studies using this fNIRS system (Lloyd-Fox et al., 2019). The data were then segmented into blocks of 24 s of data consisting of 4 s of the baseline trial prior to the onset of the stimulus, the experimental stimulus trial (10 s), plus the following baseline (10 s). To account for baseline drifts attributable to potential build-up of activation from trial to trial and/or slow fluctuations that could be of physiological origin, each block of attenuation data was de-trended with a linear fit between the average of the first 4 s and the average of the last 4 s. The attenuation data were then converted into changes in concentration in HbO_2_ and HHb using the modified Beer–Lambert law (Delpy et al., 1988) with an assumption of an age-appropriate differential pathlength factor of 5.13 (Duncan et al., 1995). A second level of automatic artifact detection and rejection was then conducted on a trial-by-trial level (within each channel) to identify excessive movement artifacts (Lloyd-Fox et al., 2019, 2010). Trials were removed if there were concentration changes greater than ±3 μmol during the 4-s baseline prior to the onset of the experimental stimuli, or if changes exceeded ±5 μmol during the experimental trial itself. These thresholds were set at different levels to ensure the rejection of abrupt changes in signal caused by motion while taking into account changes in hemoglobin levels caused by experimental conditions. This second step was designed to identify isolated trials with artifacts, for example, as caused by a sudden movement by the infant, which may not have triggered identification at the channel inspection stage of the intensity signal.

An average hemodynamic response curve was computed for each participant in each condition (English, French, BSL, and LSFB) based on valid channels in valid trials. Experimental conditions with fewer than three valid trials were excluded (i.e., trials passing channel rejection and looking time criteria). If one experimental condition was excluded (e.g., English), the other condition of the same modality was also excluded (e.g., French). Given these criteria, spoken language familiarity analyses were based on 14 monolinguals, 18 unimodal bilinguals, and 13 bimodal bilinguals (see Supplementary Table 2 for number of trials). Sign language familiarity analyses were based on eight bimodal bilinguals. Infants were included in modality analyses if they had at least three valid trials for each modality: spoken language (English + French) and sign language (BSL + LSFB). Using these criteria, modality analyses were based on 19 monolinguals, 20 unimodal bilinguals, and 21 bimodal bilinguals (see Supplementary Table 2 for number of trials).

A grand average including all infants was computed for each channel during the presentation of each language modality (spoken and signed language) and used to select an analysis time window from 8 to 16 s poststimulus onset (see Figure 1E). This period was selected to include the range of maximal concentration changes observed for HbO_2_ and HHb based on visual inspection of the current data and was informed by data analysis approaches using a similar paradigm in previous cohorts (Lloyd-Fox et al., 2013, 2009; Lloyd-Fox, Blasi, Mercure, Elwell, & Johnson, 2012). The maximum amplitude variation from baseline was generally observed at around 12 s. This is later than in most adult studies (Cutini, Moro, and Bisconti, 2012), but similar to previous infant studies with stimuli of this length and complexity (Lloyd-Fox et al., 2019, 2012). Peak amplitude variation from baseline was calculated within this time window for each infant in each experimental condition for HbO_2_ and HHb and compared statistically using twotailed *t* tests. Given the relatively large width of the time window and potential individual variability in peak latency, peak amplitudes were analyzed instead of mean amplitudes. To resolve statistical problems of multiple comparisons for these group analyses we applied the false discovery rate (FDR) correction (Benjamini & Hochberg, 1995) based on 46 channels. Given the exploratory nature of this first step in the analyses, we report our findings both before and after correction. Both a significant increase in HbO_2_ and significant decrease in HHb are commonly accepted as indicators of cortical activation in infant studies (Lloyd-Fox et al., 2010). In instances where HbO_2_ and HHb increased or decreased at the same time, the signal was considered inconsistent with a hemodynamic response (Lloyd-Fox et al., 2010; Obrig & Villringer, 2003) and was therefore not reported as a significant result.

### ROI Selection

Two a priori ROIs corresponding to classical language areas were defined: the inferior frontal region and posterior temporal region (see Figure 1D). Both regions have been widely documented to be involved in language processing and both are activated in a wide variety of language tasks in adults including prelexical phonemic processing, word retrieval and articulation, as well as processing semantic and syntactic ambiguity in adults (Price, 2010). Channels for each of these regions were selected based on anatomical coregistration of fNIRS data with individual MRI scans from a group of infants in the same age range (4–7 months; Lloyd-Fox, Richards, et al., 2014). The inferior frontal ROI included eight channels (four per hemisphere) previously coregistered within the inferior frontal lobe in 90%–100% of 4- to 7-month-olds. The posterior temporal ROI was composed of eight channels (four per hemisphere) covering the posterior temporal area and temporoparietal junction. Of these eight channels, four channels (two per hemisphere) were coregistered in the temporal lobe in 90%–100% of 4- to 7-month-olds, with the middle and superior temporal gyrus as the identified macroanatomical structures for each channel. Two channels (one per hemisphere) were coregistered in the parietal, frontal, or temporal area, with the superior temporal gyrus or postcentral gyrus as the most frequent identified macroanatomical structures. The last two channels (one per hemisphere) were adjacent, but not included in the co-registration with MRI of Lloyd-Fox, Richards, et al. (2014), as they were not part of the headgear used in their study. These channels were located over the temporoparietal junction and are most likely to overlay the posterior part of the superior temporal gyrus or the supramarginal gyrus.

### Multivariate Pattern Analyses

To compare brain activation for different experimental conditions at the network level, including the inferior frontal and posterior temporal ROIs MVPAs were used. To test for hemispheric asymmetries, patterns of activation were compared across all channels, and then within each hemisphere. Classification was conducted with a linear support vector machine packaged in MATLAB using a soft margin with the default C value of 1 for binary classifications. The patterns submitted to the analysis were the maximum amplitude in the predefined time window from the average of all trials for each participant and each experimental condition, such that each participant contributed a single pattern to each analysis (e.g., spoken vs. signed language, or familiar vs. unfamiliar languages). Channels were excluded following the same criteria described in Data Processing. Data were z-scored within each channel across all infants to ensure that features were in comparable scales for classification. We used a leave-one-participant-out approach, such that the classifier was trained on a balanced training set of neural responses from all of the participants excluding the to-be-classified participant (see Emberson et al., 2017 for a similar approach). The model was trained on the averaged pattern derived from the fNIRS epoch data for each of the two experimental conditions from all participants except one. It was then tested against the held-out pattern from the remaining participant. As such, each participant contributed two trials, one from each condition to be classified and could be classified as correctly guessed or otherwise. Hence the full data set of 60 participants yielded accuracy based on successful classifier guesses from 120 trials, and within-group analyses of 20 participants derived 40 classification trials on which accuracy was based. The classification reported is the proportion of correctly guessed trials. To ensure that classification was not biased, permutation testing was conducted by randomly permuting condition labels for each participant, such that the labels were either randomly maintained or swapped prior to training and testing of the classifier. This ensured that the participant structure was preserved in the label shuffling. For binary classifications (i.e., spoken vs. signed language or familiar vs. unfamiliar languages), 1,000 permutations were conducted and a probability value was ascertained by generating a null distribution and identifying the number of observed values that was greater than or equal to the accuracy derived from the nonshuffled data (Pereira, Mitchell, & Botvinick, 2009). The observed value was included in both the numerator and denominator for calculating the *p* value, such that if the classification accuracy observed from the data was higher than all the observed permutation values, this would result in a value of *p* = 1/1001 (Ruxton & Neuhäuser, 2013). Between-group and between-hemisphere analyses were conducted by ascertaining a null difference distribution by subtracting values derived from two null distributions. The *p* value of these analyses represents the fraction of the sample that is greater than or equal to the accuracy actually observed when using the correct labels.

## Results

To establish a full picture of the neural activation in response to each language modality, we begin by presenting channel-by-channel analyses for the spoken and the signed language modalities in all infants taken as a whole, as well as in each group of infants. These are followed by channel-by-channel analyses of the impact of language familiarity on these patterns of activation. Next, we focus on frontotemporal ROIs to statistically compare the activation within these language areas of the left and right hemispheres, between groups of infants, in response to each language modality and language familiarity. Finally, we present MVPAs aiming to contrast language modalities and language familiarity at the network level.

### Univariate Analyses

#### Language modality

**Figure 2:**
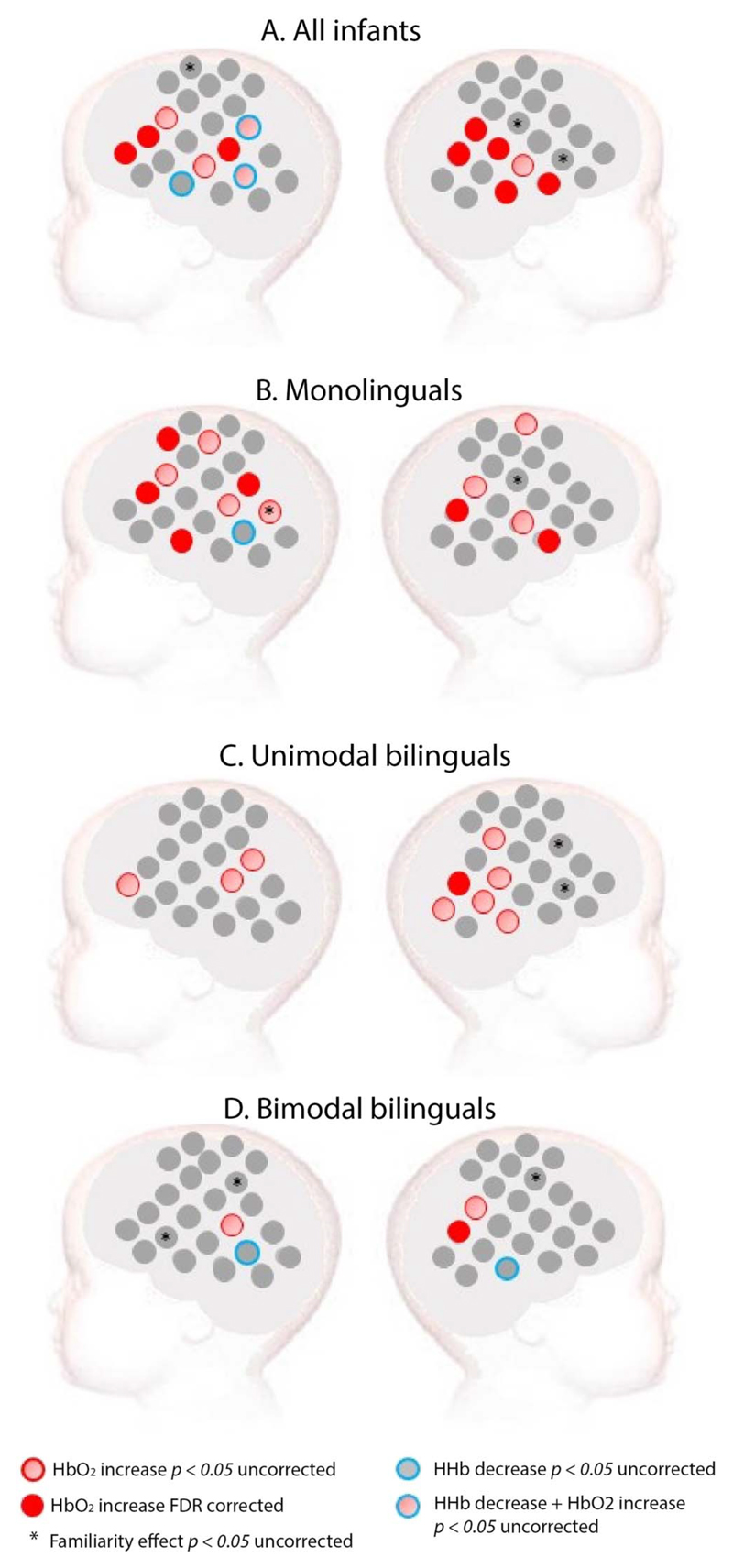
Activation for spoken language in (A) all infants, (B) monolinguals, (C) unimodal bilinguals, and (D) bimodal bilinguals. *Red*: Channels showing a significant increase in HbO_2_ in channel-by-channel analyses at the FDR-corrected level. *Pink*: Channels showing a significant increase in HbO_2_ in channel-by-channel analyses at an uncorrected statistical level of *p* < 0.05. *Blue*: Channels showing a significant decrease in HHb at an uncorrected statistical level of *p* < 0.05. No channel showed a significant HHb effect at an FDR-corrected level. *Stars* indicate a significant difference between a familiar (English) and an unfamiliar (French) spoken language at an uncorrected statistical level of *p* < 0.05.

When all 60 infants were considered as a single group, widespread activation was observed in response to spoken language versus baseline. Significant increase of HbO_2_ was found in a bilateral network that included the temporal and the inferior frontal areas of the brain (see Figure 2). When monolingual infants were considered alone, a significant increase of HbO_2_ was found in a bilateral network that included the temporal and the inferior frontal areas of the brain (see Supplementary Figure 3 for grand averaged hemodynamic response in each channel, group, and experimental condition). Unimodal bilinguals showed bilateral activation with an uncorrected *p* value, but the only channel surviving FDR correction was located in the right posterior temporal area. Bimodal bilinguals had fewer active channels at an uncorrected *p* value compared to the other two groups, especially in the left inferior frontal region. The only channel surviving FDR correction in bimodal bilinguals was in the right posterior temporal area. None of the channels demonstrating a significant decrease in HHb survived FDR correction.

**Figure 3:**
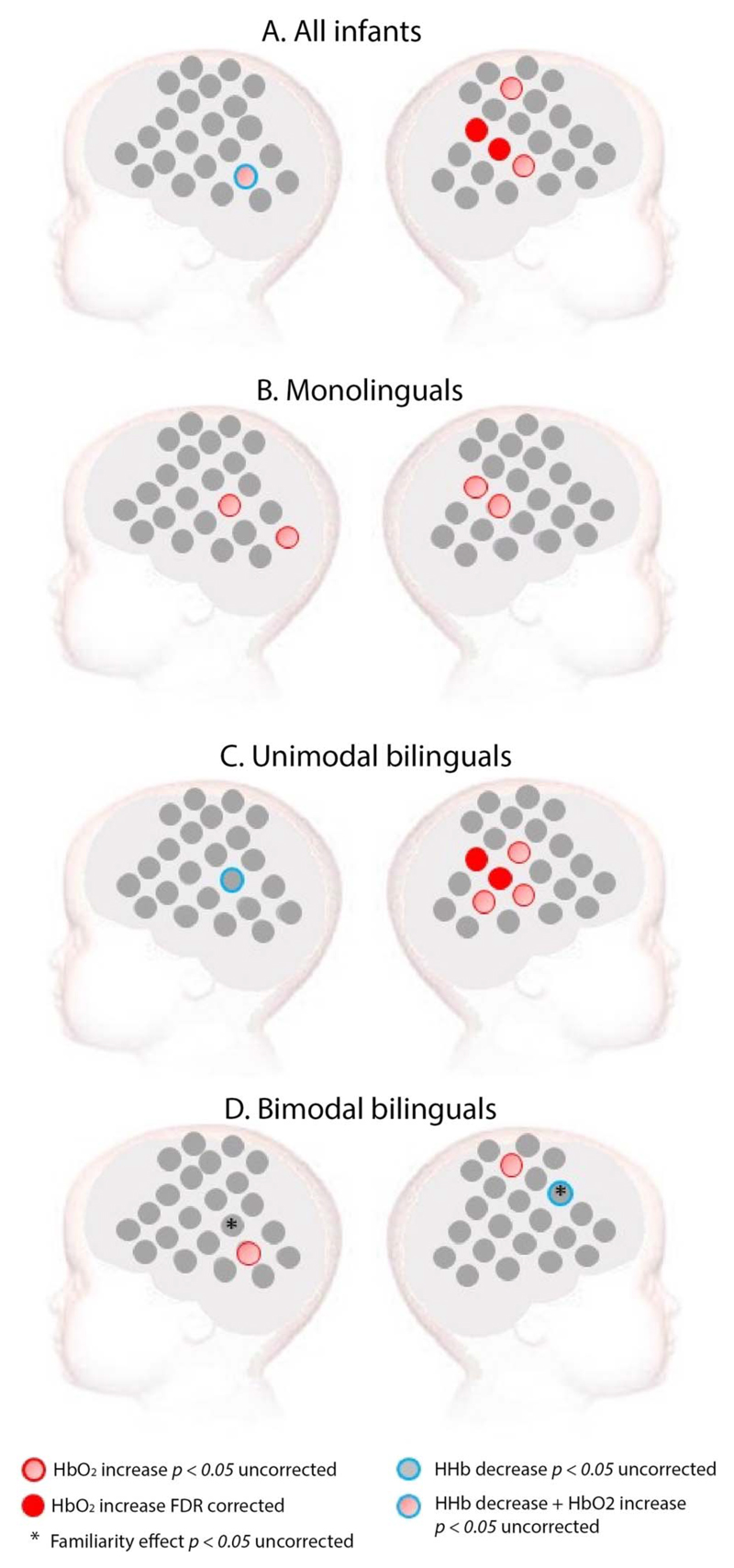
Activation for sign language in (A) all infants, (B) monolinguals, (C) unimodal bilinguals, and (D) bimodal bilinguals (D). *Red*: Channels showing a significant increase in HbO_2_ in channel-by-channel analyses at the FDR-corrected level. *Pink*: Channels showing a significant increase in HbO_2_ in channel-by-channel analyses at an uncorrected statistical level of *p* < 0.05. *Blue*: Channels showing a significant decrease in HHb at an uncorrected statistical level of *p* < 0.05. No channel showed a significant HHb effect at an FDR-corrected level. *Stars* indicate a significant difference between a familiar (BSL) and unfamiliar (LSFB) sign language at an uncorrected statistical level of *p* < 0.05. These familiarity effects were assessed only in the bimodal bilingual group given that both signed languages were unfamiliar to infants of the other groups.

In contrast, sign language elicited a significant increase in HbO_2_ compared to baseline, mainly in the right temporoparietal area when all infants were considered as a single group (see Figure 3). In monolinguals, sign language elicited increased HbO_2_ in the temporoparietal area of both hemispheres with an uncorrected *p* value, but no channel survived FDR correction. In unimodal bilinguals, a significant increase of HbO_2_ was observed in the right temporoparietal area. Surprisingly, in bimodal bilinguals, only a few scattered channels showed a significant response to sign language at an uncorrected *p* value, none of which survived FDR correction (see Figure 3).

These analyses suggest that spoken language elicited more widespread activation than signed language, and that the lateralization of this activation differs between groups. These observations were assessed using ROI analyses, and differences in patterns of activation between each language modality were assessed with MVPA. In accordance with previous infant research (Cristia et al., 2013; Gervain et al., 2011; Lloyd-Fox et al., 2010) the majority of the significant channel-by-channel effects were in HbO_2_ for both spoken and signed languages. No HHb effect was significant after FDR correction, so all subsequent familiarity analyses, ROI analyses, and MVPAs focus on HbO_2_.

#### Language familiarity

The peak amplitude of HbO_2_ was directly contrasted for English (familiar spoken language to all infants) and French (unfamiliar spoken language to all infants) on a channel-by-channel basis. Analyses of all infants together suggested that a few channels elicited a difference between familiar and unfamiliar spoken language. This was also the case when each group of infants was analyzed separately. However, these channels did not cluster together and none of these effects survived FDR correction (see Figure 2).

In bimodal bilinguals, we also tested for sign language familiarity effects. BSL (familiar) elicited increased activity compared to LSFB (unfamiliar) in one right frontal channel, whereas the opposite effect was found in one left temporoparietal channel (see Figure 3). However, these effects did not survive FDR correction. Familiarity effects are not reported for the other two groups of infants, since both sign languages were unfamiliar to them.

### ROI Analyses

#### Language modality

**Figure 4:**
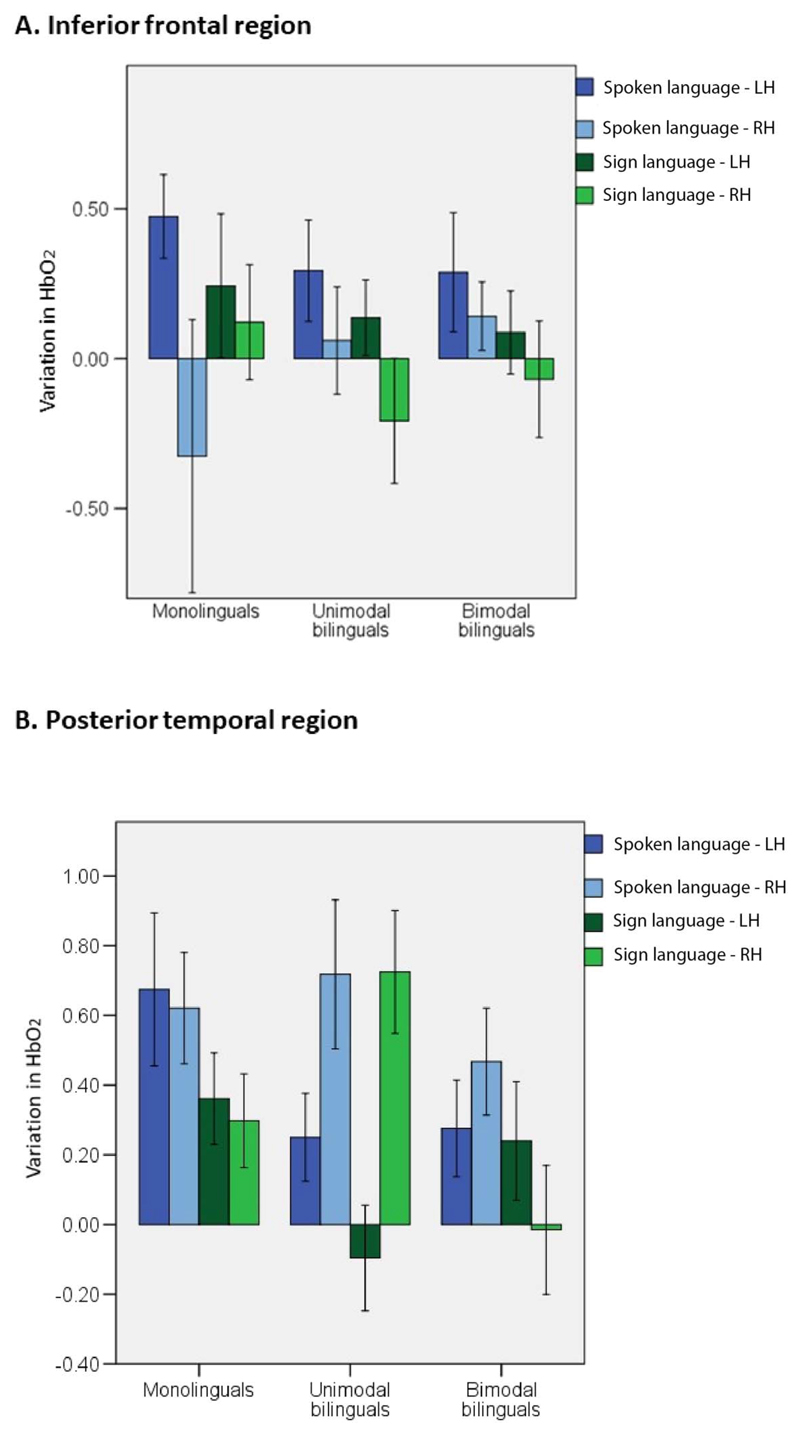
Mean variation in HbO_2_ in the inferior frontal and posterior temporal ROIs for each language modality in each group of infants. Error bars represent standard error.

To gain a better understanding of the activation within language areas, two ROI analyses were defined corresponding to the inferior frontal and posterior temporal areas. Peak HbO_2_ response was averaged for all channels within each ROI for each infant, in each hemisphere and condition. Activation in each ROI was analyzed in a 2 × 2 × 3 ANOVA with language modality (spoken, signed) × hemisphere (left, right) × group (monolinguals, unimodal bilinguals, bimodal bilinguals). In the inferior frontal ROIs (see Figure 1D), there was a significant effect of hemisphere, *F*(1, 57) = 6.3; *p* = 0.015; η^2^ = 0.100, but no other effect or interaction reached significance level (see Figure 4). Activation in the inferior frontal region was left-lateralized regardless of group and language modality. This result suggests that activation within the inferior frontal ROI is not specific to spoken language and not significantly influenced by an infant’s experience of different language modalities. We note with interest the increased variability in right hemisphere activation in the inferior frontal region to spoken language in the monolingual group. Although left hemisphere activation was strong in most monolingual infants, a strong right hemisphere deactivation was observed in many monolingual infants in response to spoken language. It may be that this right hemisphere deactivation develops with functional specialization for language. The monolingual group may be more advanced in the process of functional specialization for language because of reduced variability in language input. Right hemisphere deactivation to spoken language may be most reliably observed in older infants and as language proficiency develops. These speculative interpretations require further investigation, ideally in a group of infants with a smaller age range.

In the posterior temporal region, there was a significant effect of modality, *F*(1, 55) = 6.8; *p* = 0.012; η^2^ = 0.109. This region activated more in response to spoken than signed language. There was also a significant interaction between hemisphere × group, *F*(2, 55) = 6.8; *p* = 0.002; η^2^ = 0.199. Separate ANOVAs in each group revealed that activation in the posterior temporal area was right lateralized in unimodal bilinguals in response to spoken and signed language, *F*(1, 17) = 9.8; *p* = 0.006; η^2^ = 0.365, whereas monolinguals, *F*(1, 18) = 0.2; *p* = 0.653; η^2^ = 0.011, and bimodal bilinguals, *F*(1, 20) = 0.1; *p* = 0.778; η^2^ = 0.004, showed no difference in left and right activation. This result suggests that the experience of two spoken languages in infancy influences lateralization of activation in response to language of any modality within the posterior temporal ROI.

#### Language familiarity

In addition, analyses within each ROI were performed to assess for familiarity effects within each modality. For spoken language, a 2 × 2 × 3 ANOVA with familiarity (familiar, unfamiliar) × hemisphere (left, right) × group (monolinguals, unimodal bilinguals, bimodal bilinguals) did not reveal any familiarity effects in the temporal or inferior frontal ROIs (all *p* > 0.14; η^2^ < 0.001 for the inferior frontal area and η^2^ = 0.005 for the posterior temporal area). For sign language, familiarity effects were assessed only within bimodal bilinguals, as both language modalities were unfamiliar to infants of other groups. No familiarity effect was significant in either ROI (all *p* > 0.14; η^2^ = 0.147 for the inferior frontal area and η^2^ = 0.149 for the posterior temporal area). This analysis may have been underpowered given that only eight infants survived inclusion criteria. A power analysis reveals that 12 participants would have been required to achieve 90% power.

### Multivariate Pattern Analyses

#### Language modality

**Figure 5:**
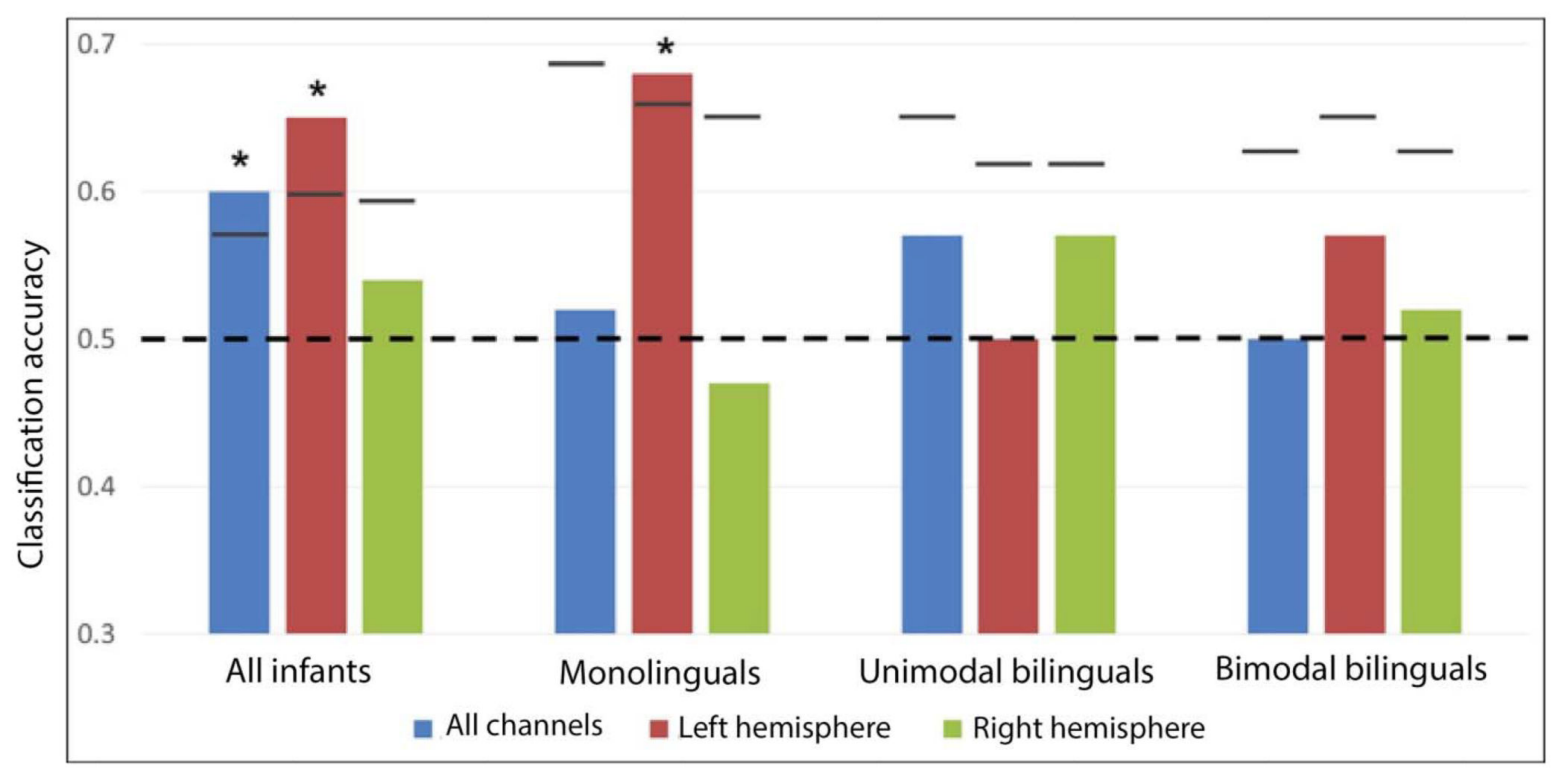
Classification accuracy for spoken versus signed language. Empirical proportion of correct classification for spoken versus signed language by MVPAs. Empirical proportion of correct classification is presented for all channels, left hemisphere channels, and right hemisphere channels in each group of infants. The *dotted line* represents chance level (0.5), *short lines* represent the upper bound of the 95% confidence interval, and *stars* represent classification models that are significantly more successful than chance.

Patterns of activation for spoken and signed language were compared. Each participant’s average maximum amplitude of HbO_2_ in the predefined time window in response to spoken language (English + French) and signed language (BSL + LSFB) was entered in MVPAs. Data from all channels were analyzed (dimensions of feature vector = 46) and then separate analyses were performed for each hemisphere (feature vector = 23) to test for hemispheric differences. When all 60 infants were taken as a single group (see Figure 5 for MVPA results and Supplementary Figure 2 for a channel-by-channel comparison of language modalities), patterns of activation for spoken and signed language could be classified at a level greater than chance using all 46 channels (proportion correct = 0.60; permutation *p* value = 0.029), or left hemisphere channels (proportion correct = 0.65; permutation *p* value = 0.010), but not right hemisphere channels (proportion correct = 0.54; permutation *p* value = 0.270). The difference in classification accuracy between the left and right hemispheres was not significant (*p* = 0.218).

In monolinguals, patterns of activation for spoken and signed language could be classified at a level greater than chance using left hemisphere channels (proportion correct = 0.68; *p* = 0.039), but not right hemisphere channels (proportion correct = 0.47; *p* = 0.733) or all channels (proportion correct = 0.52; *p* = 0.520). The difference in classification accuracy between the left and right hemispheres was not significant (*p* = 0.118). It was predicted that classification would be less successful in bimodal bilinguals compared to monolinguals and unimodal bilinguals given that these infants have experience in both language modalities. However, MVPAs could not classify spoken and signed language with an accuracy greater than chance in either group of bilinguals using all channels (all *p* > 0.2), left channels (all *p* > 0.5), or right channels (all *p* > 0.2; see Supplementary Table 3). Furthermore, although classification based on left hemisphere channels was successful in only monolinguals, when classification accuracies in the left hemisphere were compared between groups, this difference was not statistically significant (monolinguals vs. unimodal bilinguals [*p* = 0.123]; monolinguals vs. bimodal bilinguals [*p* = 0.406]).

These results suggest that spoken and signed language modalities are associated with differentiated brain activation patterns in monolingual infants, but not in both groups of bilinguals. However, the strength of this conclusion is constrained by the absence of a difference in accuracies between groups.

#### Language familiarity

Patterns of activation for familiar and unfamiliar spoken languages were compared at the network level using MVPAs. The average of maximum amplitude of HbO_2_ of all trials in the pre-defined time window for each participant in response to spoken English (familiar) and spoken French (unfamiliar) was entered in MVPAs. Classification was not successful in any group based on all channels, left or right hemisphere channels (see Supplementary Table 4). Classification of familiar (BSL) and unfamiliar sign languages (LSFB) in bimodal bilinguals was not significantly more successful than chance (see Supplementary Table 4). These group analyses were based on small samples and may lack power.

### Discussion

The present study investigated the neural activation for spoken and signed language in three groups of infants with very different language experiences. Although the three groups had different experience of speech, language, and communication, they shared the experience of normal hearing and the exposure to English as a familiar spoken language. When this group of 60 infants was considered as a whole, we found activation in response to audiovisual infant-directed spoken language in a wide bilateral network, which included the posterior temporal and the inferior frontal areas. This pattern of activation is similar to the network involved in spoken language processing in adults (Price, 2010) and also to that previously reported in infants (Altvater-Mackensen & Grossmann, 2016; Dehaene-Lambertz et al., 2002, 2006, 2010; May et al., 2018; Minagawa-Kawai et al., 2010; Pena et al., 2003; Perani et al., 2011; Sato et al., 2012; Shultz et al., 2014; Vannasing et al., 2016). Sign language elicited activation in a few channels located in the right temporoparietal area. Multivariate analyses suggest that spoken and signed language elicited different patterns of neural activation, which could be decoded with an accuracy greater than chance based on all channels and based on left hemisphere channels, but not based on right hemisphere channels. This suggests that the left hemisphere is more sensitive to language modality than the right hemisphere in infancy, even though only one third of the infants had prior experience of sign language as a mode of communication.

It is important to note that these patterns of activation emerge from differences in brain oxygenation between experimental conditions and baseline. During these baseline intervals, infants were presented with a variety of static pictures to keep their attention to the screen. These consisted of pictures of animals, babies, and modes of transportation. Any difference in activation between experimental conditions and baseline may be triggered not only by the presence of language (spoken or signed language), but also by biological motion and/or the consistent presence of faces in both language conditions. In that respect, we can interpret these patterns of activation as associated with the audiovisual communicative experience of language.

Having established the pattern of activation in response to spoken and signed language across the whole group of infants, we examined our specific aims of clarifying the role of language experience in shaping brain activation for language. We compared brain activation for spoken and for signed language and for familiar and unfamiliar languages in each group of infants.

#### Language Modality

In monolinguals, activation for spoken language compared to baseline was found in a large bilateral network of channels including the inferior frontal and posterior temporal regions, whereas sign language did not elicit any activation compared to baseline, which survived correction for multiple comparisons. In unimodal bilingual infants, both spoken and signed language elicited activation in the right temporoparietal area. Bimodal bilinguals showed activation to spoken language in the right posterior temporal area, while sign language did not elicit any activation that survived correction for multiple comparisons.

Our original predictions were that reduced experience of spoken language in bimodal bilinguals compared to monolinguals and unimodal bilinguals would be associated with reduced amplitude and lateralization of activation in frontotemporal language areas for spoken language. Conversely, we predicted that the bimodal bilingual’s experience of sign language would lead to increased amplitude of activation in frontotemporal language areas and increased lateralization for sign language. Activation in the inferior frontal region of interest was left lateralized regardless of group and language modality. Therefore, left lateralized activation in this region in infancy is not specific to spoken language, and can also be observed for sign language regardless of the infant’s experience of this language modality. It is possible that left lateralized activation is elicited in the inferior frontal area in response to stimuli with a complex structure such as that of spoken or signed language, and/or by stimuli of a social communicative nature.

In contrast, there was greater activation in the posterior temporal ROI for spoken than for signed language regardless of hemisphere. Moreover, lateralization within this region significantly differed between groups of infants. Contrary to our prediction, this significant effect was not driven by bimodal bilinguals, but by unimodal bilinguals who showed right lateralization in this area in response to both spoken and signed language. Activation in the posterior temporal ROI was not lateralized for spoken and signed language in both monolinguals and bimodal bilinguals. Previous studies with proficient unimodal bilingual adults suggest that both languages engage similar neural regions, located primarily in the left hemisphere (Abutalebi, Cappa, & Perani, 2001; Liu & Cao, 2016; Perani et al., 1998). Some studies comparing monolingual and unimodal bilingual adults who acquired their languages early have revealed an increased participation of the right hemisphere during language processing in bilinguals (Hull & Vaid, 2007; Polczyńska, Japardi, & Bookheimer, 2017). The present study extends these results to show increased right lateralization in preverbal unimodal bilinguals during the processing of infant-directed spoken language. Moreover, this pattern of increased right hemisphere engagement extended to the processing of sign language in unimodal bilingual infants. That is, experiencing two spoken languages from birth influences brain activation in response to sign language in unimodal bilinguals, even when experienced for the very first time.

It is possible that the unimodal bilingual experience in infancy contributes to developing an increased sensitivity to the general structure of languages, which in turn would lead to similar patterns of activation for an unfamiliar language modality in this group. Indeed, both spoken and signed languages are formed of a hierarchical structure where phonological, semantic, and syntactic elements are combined to produce utterances (Sandler & Lillo-Martin, 2006). It is also possible that this similarity in structure leads to a similar pattern of activation for both language modalities.

In addition, unimodal bilinguals may have an increased sensitivity to visual speech articulation. This could explain why unimodal bilingual infants are better than monolinguals at discriminating two foreign spoken languages based on silent articulation (Sebastián-Gallés, Albareda-Castellot, Weikum, & Werker, 2012). Other studies also demonstrated increased attention capture and maintenance by face stimuli in unimodal bilingual infants compared to both monolinguals and bimodal bilinguals (Mercure, Kushnerenko, et al., 2018; Mercure, Quiroz, et al., 2018). The stimuli used in the present study contained some English lip movements often present in natural BSL. It is therefore possible that the activation for sign language in unimodal bilingual infants is in part associated with the processing of these visual cues of articulation. Sign language stimuli without any lip movements or with the mouth obscured would be required to evaluate this possibility.

Perhaps surprisingly, our data suggest that compared to the bimodal bilingual experience, the unimodal bilingual experience appears to have more impact on lateralization of activation for language perception. Spoken languages are all produced by movements of the vocal tract and perceived as audiovisual speech. In that respect, they are more similar than a spoken and signed language. Learning two similar but not identical codes may require increased cognitive resources in order to differentiate them, compared to learning a spoken and a signed language. Spoken and signed languages are more easily differentiated and can also be produced simultaneously, often referred to as code-blending (Emmorey, Luk, Pyers, & Bialystok, 2008). In that respect, a unimodal bilingual experience in infancy may have a stronger impact than a bimodal bilingual experience on the process of neural specialization in infancy. Unimodal bilingual infants may focus on the rhythm of the speech input to support the differentiation of spoken languages, while the differentiation of spoken and signed languages is perceptually more salient. This increased focus on speech rhythm may explain the increased right hemisphere involvement in unimodal bilinguals compared to other groups of infants (Riecker, Wildgruber, Dogil, Grodd, & Ackermann, 2002).

Bimodal bilinguals demonstrated a smaller number of active channels in response to both language modalities compared to monolinguals and unimodal bilinguals. They also tended to have smaller amplitudes of activation in both ROIs (the inferior frontal and posterior temporal regions) compared to monolinguals. However, no group effects were found on the mean amplitude of the response to spoken and signed language in these ROIs. Although this pattern of results may provide some weak support for the hypothesis of reduced amplitude of activation for spoken language in this group, the trend also applied to sign language, which was contrary to our hypotheses. Of interest, this trend for reduced activation in response to both language modalities was observed in the absence of a receptive language deficit in this group. To the contrary, bimodal bilingual infants outperformed the other two groups of infants on communicative skills, as measured by the receptive language scale of the Mullen Scales of Early Learning. This is interesting given that this scale was designed with hearing infants of hearing parents in mind and included items that may not be as familiar to infants with deaf parents, such as calling their names from behind their back. It is interesting to note that sighted infants with blind parents have also been shown to outperform sighted infants of sighted parents on visual receptive skills (Senju et al., 2013). Like sighted infants with blind parents, bimodal bilinguals need to adapt their communicative strategies to communication partners with different needs. This flexibility may lead to more mature skills in some aspects of their development. That bimodal bilinguals had better communicative skills than the other two groups, as measured by the receptive language scale of the Mullen Scales of Early Learning, yet showed very few active channels in response to spoken and signed language, could suggest that reduced or more focal language activation may be associated with better communicative skills. However, future studies with larger samples are required to test this possibility. It would also be interesting to see whether the same association is observed with more language-specific abilities, such as the development of vocabulary and syntax later in childhood.

Multivariate analyses suggested that spoken and signed language elicited different patterns of activation in monolinguals, which could be classified at an accuracy greater than chance based on left hemisphere activity. The classification accuracy (68%) was reasonably high, especially given that it was performed between participants. This accuracy is comparable to trial-level decoding found in adult fMRI studies (Evans et al., 2013; Formisano, De Martino, Bonte, & Goebel, 2008; McGettigan et al., 2012; Misaki, Kim, Bandettini, & Kriegeskorte, 2010). This is also similar to the accuracy found in fNIRS studies in between-infant decoding (Emberson et al., 2017; data set no. 2, mean = 72%) and between-subject decoding in adults (Zinszer, Bayet, Emberson, Raizada, & Aslin, 2017; mean = 70%). On the other hand, classification was not successful in any of the two groups of bilinguals. Both groups of bilinguals have more diversity in their spoken language experience compared to monolinguals. Not only is their experience characterized by more than one language, but it is also likely to include more variable models within a language, including foreign accents, and/or less precise phonological distinctions produced by deaf adults (Lane & Webster, 1991; Waldstein, 1990). This increased variability in input could influence the process of neural specialization for language in these bilingual infants, leading to more variable brain representation.

#### Language Familiarity

The second aim of this study was to assess the impact of language experience on the brain signature for language familiarity. A spoken language familiar to all infants was presented (English), as well as a spoken language unfamiliar to all infants (French). Based on the literature, increased activation was predicted for the familiar versus the unfamiliar language (Fava et al., 2014; Minagawa-Kawai et al., 2010; Sato et al., 2012; Vannasing et al., 2016), especially in the left hemisphere. It was also predicted that familiarity effects would be smaller in both bilingual groups compared to monolinguals because of their reduced exposure to the familiar language. In each group of infants, a few channels showed differential activation for the familiar language compared to the unfamiliar language, but none of these channels clustered and no familiarity effects survived FDR correction. Moreover, ROI analyses revealed no effect of familiarity and no interaction of familiarity with group or hemisphere. MVPAs could not classify familiar and unfamiliar spoken languages based on distributed patterns of activation in all infants or in any of the infant groups. The difference in the patterns of brain activation for a familiar and an unfamiliar language in preverbal infants may not have sufficient stability across participants to allow categorization. Moreover, infants included in this study had a wide age range that is associated with various stages in the process of language-specific specialization (Fava et al., 2014). This could explain the lack of significant familiarity effects at the group level in the present study.

A familiar sign language was also expected to elicit increased activation and lateralization in comparison with an unfamiliar sign language in bimodal bilinguals. This hypothesis was not supported by the data, as no effects of sign language familiarity were observed. Familiar and unfamiliar sign languages could not be classified based on MVPA in bimodal bilinguals. Only eight infants reached the inclusion criteria to allow comparison of the two sign language conditions. For this reason, this analysis may be underpowered and these null results should be interpreted cautiously.

#### Summary

The results of the present study suggest that the neural substrates of language are influenced by early experience of language. Contrary to our predictions, our primary finding was related to the patterns observed in unimodal bilingual infants. These hearing babies exposed to two spoken languages from birth, appear to engage the right hemisphere to a greater extent than the left during perception of spoken language and also sign language, of which they had no previous experience. Unimodal bilingual experience may lead to increased sensitivity to the structure of languages and/or to increased sensitivity to lip patterns that often accompany sign language. Patterns of activation for spoken and signed language could be successfully classified in monolinguals, but not in any of the bilingual groups. Bimodal bilinguals demonstrated a tendency for less activation in response to both language modalities; however, this trend was accompanied by an increase in behaviorally measured communicative skills. Our results indicate that the neural substrate of language is plastic in infancy and influenced by language input and language modality, suggesting that there may be different neural paths to language development.

## Supplementary Material

Supplementary Material

## Figures and Tables

**Table 1:** Mullen Scales of Early Learning *t* scores in the receptive language, visual reception, fine motricity, and gross motricity scales in each group

	Monolinguals (n = 19)	Unimodal bilinguals (n = 20)	Bimodal bilinguals (n = 21)
Receptive language	44.2 (12.8) [20–64]	43.5 (11.9) [20–63]	52.9 (10.5) [20–66]
Visual reception	53.5 (12.2) [26–80]	46.8 (11.1) [27–64]	51.9 (12.1) [32–76]
Fine motricity	51.7 (9.4) [26–64]	46.6 (9.9) [27–64]	51.6 (10.4) [26–68]
Gross motricity	56.0 (10.8) [31–77]	52.8 (8.1) [40–68]	49.5 (9.9) [30–67]

Group mean (standard deviation), [range].
