# Supplementary material for: Language Experience Impacts Brain Activation for Spoken and Signed Language in Infancy: Insights From Unimodal and Bimodal Bilinguals
Source: Neurobiol Lang (Camb). Author manuscript; Available in PMC 2020 Apr 9. (PMC7145445; doi:10.1162/nol_a_00001)
Supplement: Supplementary file 1 [file EMS85856-supplement-Supplementary_Material.pdf]

Language experience impacts brain activation for spoken and signed language in infancy:

Insights from unimodal and bimodal bilinguals

SUPPLEMENTARY MATERIAL

Evelyne Mercure [1, 2, 3], Samuel Evans [2, 4], Laura Pirazzoli [3, 5], Laura Goldberg [2], Harriet Bowden-Howl [2, 6], Kimberley Coulson-Thaker [2, 7], Indie Beedie [2], Sarah Lloyd-Fox [3, 8], Mark H Johnson [3, 8], Mairéad MacSweeney [2]

[1]Goldsmiths, University of London, [2]University College London, [3]Birkbeck - University of London, [4] University of Westminster, [5]Boston Children's Hospital, [6]University of Plymouth, [7]University of Hertfordshire, [8]University of Cambridge

Additional languages/ Language combinations	Number of infants
Polish	3
Bengali	2
Spanish	2
Greek	2
Italian	1
German	1
Cantonese	1
Norwegian	1
Portuguese	1
Ukrainian	1
Russian	1
Italian, Cantonese	1
German, Romanian	1
Russian, Portuguese	1
Farsi, Gujarati, Swahili	1

Supplementary Table 1: Addition languages or language combinations that unimodal bilinguals were exposed to in addition to English

	Spoken languages	Sign languages	English	French	BSL	LSFB
**Monolinguals**	8.2 (2.0)<br><br>[n = 19]	7.8 (2.0)<br><br>[n = 19]	4.5 (0.6)<br><br>[n = 14]	4.6 (0.6)<br><br>[n = 14]		
**Unimodal bilinguals**	8.8 (1.0)<br><br>[n = 20]	8.2 (1.7)<br><br>[n = 20]	4.4 (0.6)<br><br>[n = 18]	4.7 (0.7)<br><br>[n = 18]		
**Bimodal bilinguals**	7.4 (1.7)<br><br>[n = 20]	6.3 (2.1)<br><br>[n = 20]	4.2 (0.6)<br><br>[n = 12]	4.1 (0.7)<br><br>[n = 12]	4.3 (0.6)<br><br>[n = 8]	4.3 (1.0)<br><br>[n = 8]

Supplementary Table 2: Average number of included trials (and standard deviation) in each experimental condition and each group. The numbers in square brackets refer to the number of included participants. BSL and LSFB conditions are not reported for monolinguals and unimodal bilinguals given that these groups do not contribute to familiarity analyses.

	Language modality		
	All	Left	Right
All infants	0.60<br>***p* = 0.029**	0.65<br>***p* = 0.010**	0.54<br>*p* = 0.270
Monolinguals	0.52<br>*p* = 0.520	0.68<br>***p* = 0.039**	0.47<br>*p* = 0.733
Unimodal bilinguals	0.57<br>*p* = 0.242	0.50<br>*p* = 0.512	0.57<br>*p* = 0.210
Bimodal bilinguals	0.50<br>*p* = 0.550	0.57<br>*p* = 0.345	0.52<br>*p* = 0.502

Supplementary Table 3: Proportion of correct classifications (and permutation *p*-values) in MVPAs for spoken versus signed language. Values in bold indicate significant p-values ($p < 0.05$).

	Spoken language			Sign language		
	All	Left	Right	All	Left	Right
All infants	0.49<br>*p* = 0.752	0.48<br>*p* = 0.643	0.49<br>*p* = 0.673			
Monolinguals	0.39<br>*p* = 0.941	0.39<br>*p* = 0.921	0.64<br>*p* = 0.119	-	-	-
Unimodal bilinguals	0.39<br>*p* = 0.950	0.42<br>*p* = 0.871	0.58<br>*p* = 0.148	-	-	-
Bimodal bilinguals	0.50<br>*p* = 0.554	0.50<br>*p* = 0.624	0.31<br>*p* = 0.980	0.50;<br>*p*= 0.505	0.62;<br>*p* = 0.345	0.38;<br>*p* = 0.861

Supplementary Table 4: Proportion of correct classifications (and permutation *p*-values) in MVPAs for familiar versus unfamiliar languages.

A.

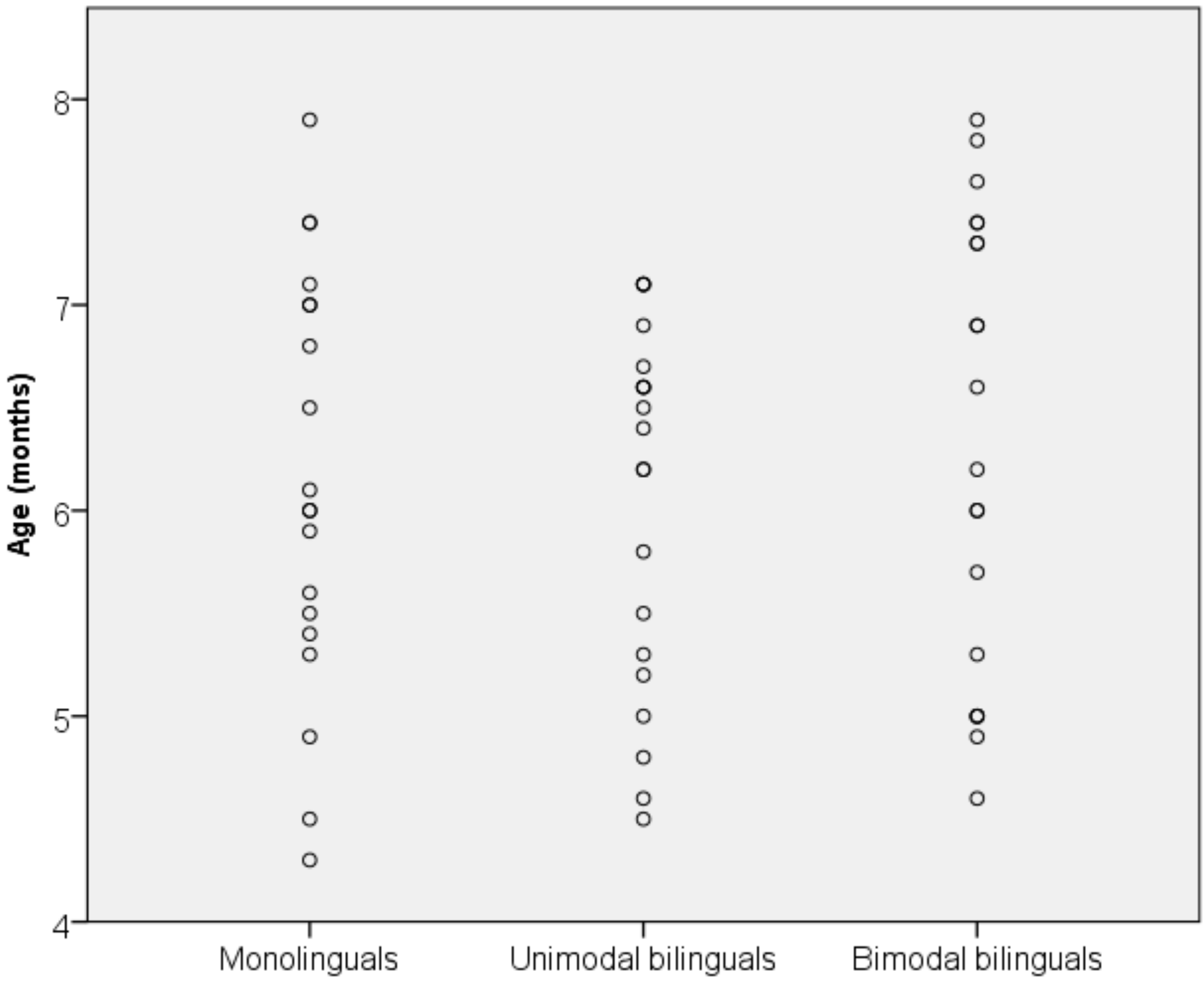

Age (months)
8
7
6
5
4
Monolinguals
Unimodal bilinguals
Bimodal bilinguals

B.

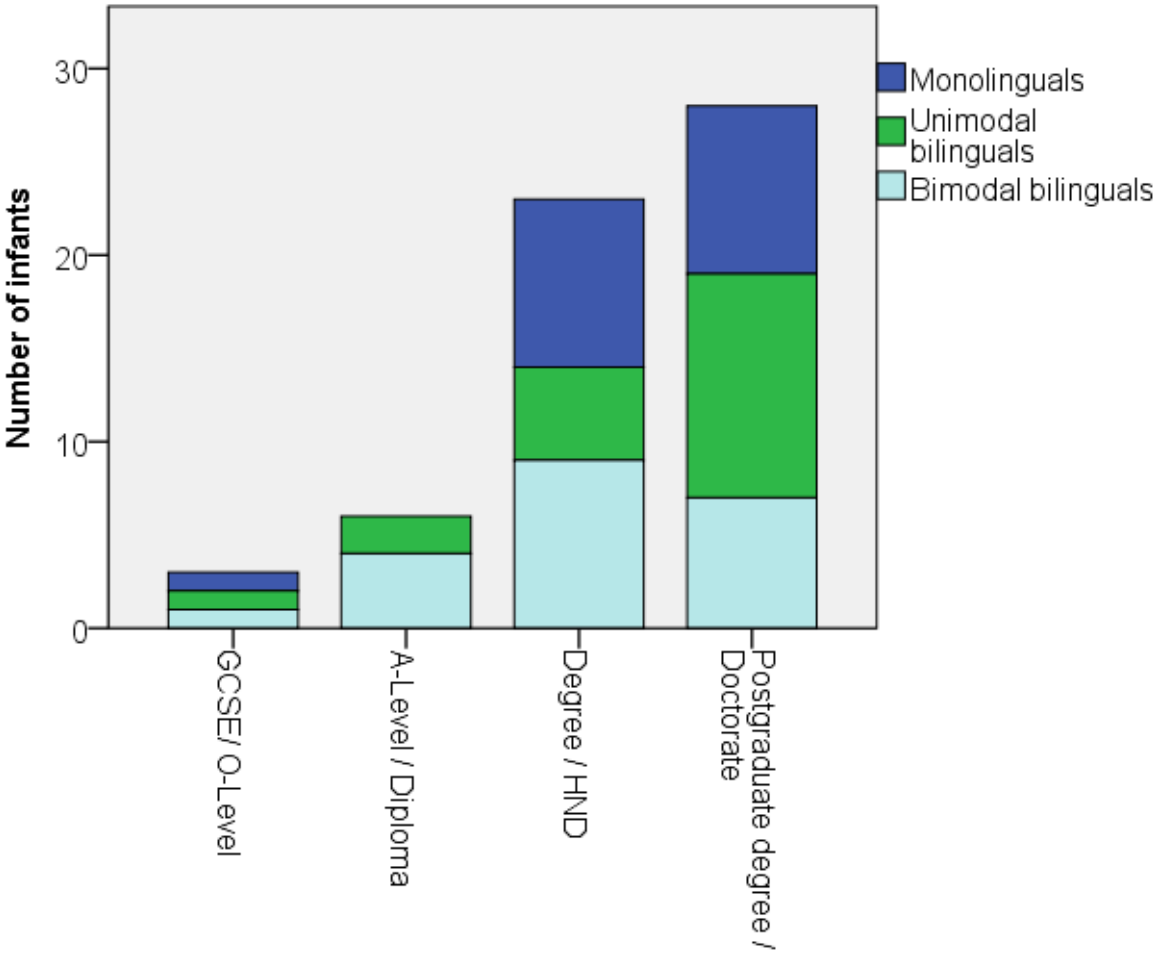
Number of infants
30
20
10
0
Monolinguals
Unimodal bilinguals
Bimodal bilinguals
GCSE/ O-Level
A-Level / Diploma
Degree / HND
Postgraduate degree / Doctorate

C.

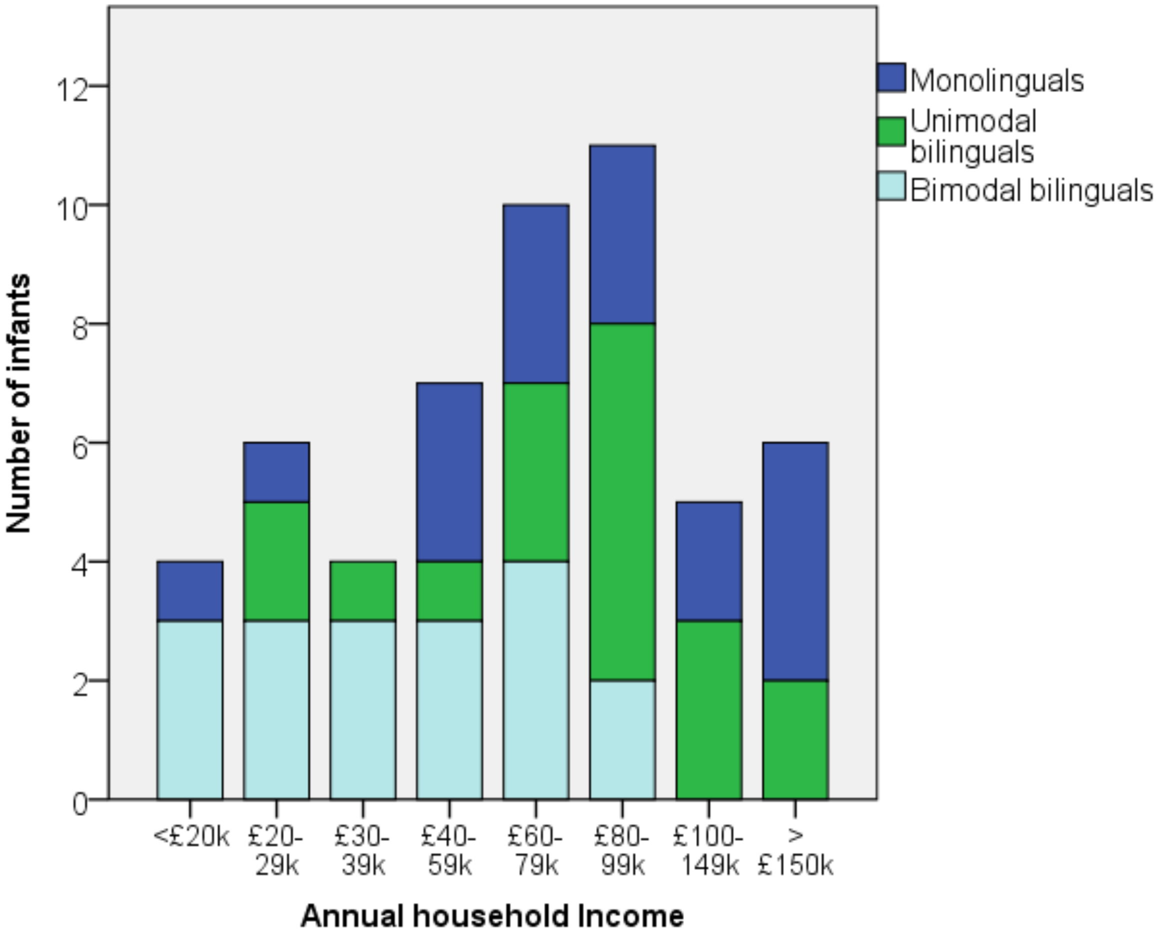

Supplementary Figure 1: Demographic details of infant participants: (A) distribution of participant's age in months, (B) maternal education, and (C) annual household income in each group of infants

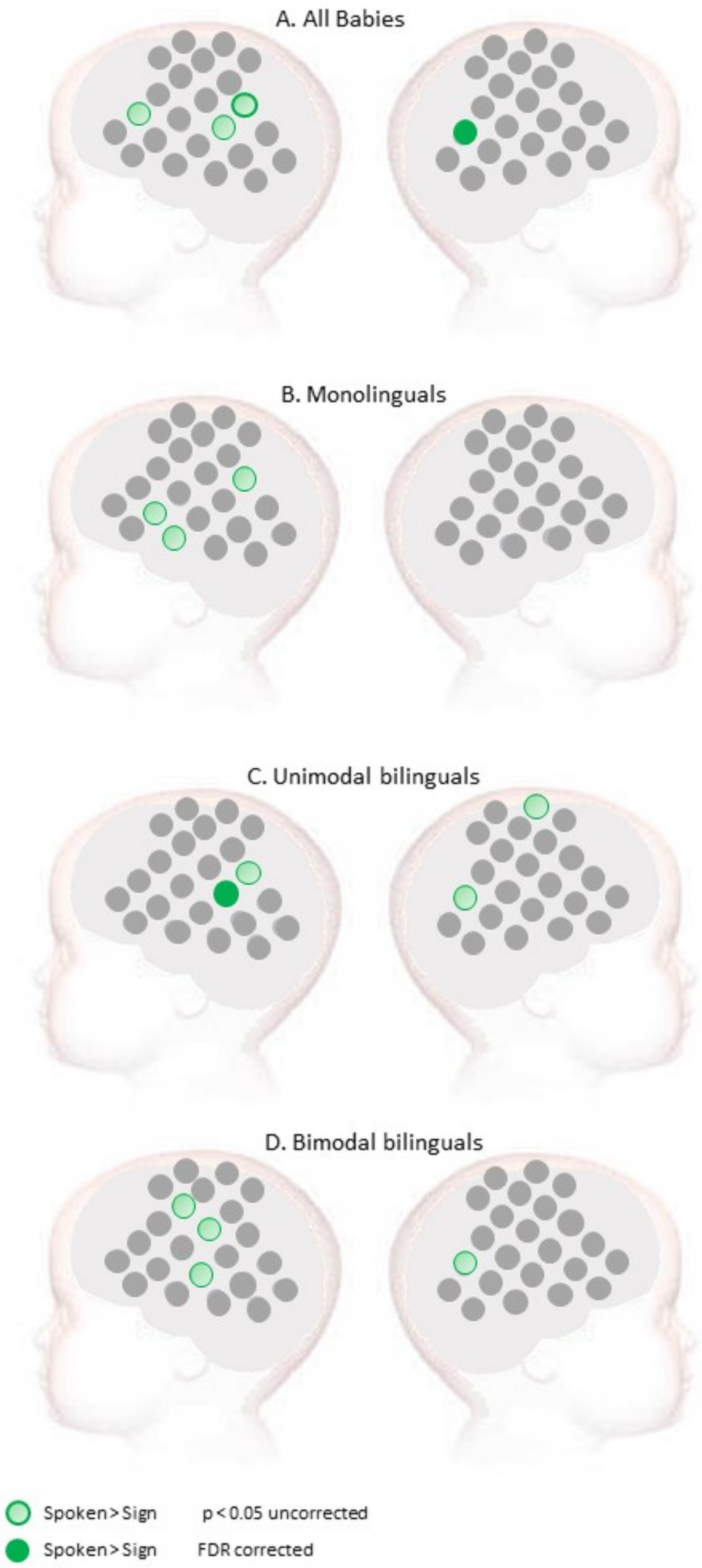

A. All Babies
B. Monolinguals
C. Unimodal bilinguals
D. Bimodal bilinguals
Spoken > Sign p < 0.05 uncorrected
Spoken > Sign FDR corrected

Supplementary Figure 2: Channel-by-channel modality contrast. Highlighted channels had significantly more activation for spoken than signed language at an uncorrected *p*-value of 0.05 (light green) or at an FDR corrected level (dark green). No channel showed a significant increase of activation for signed language compared to spoken language.

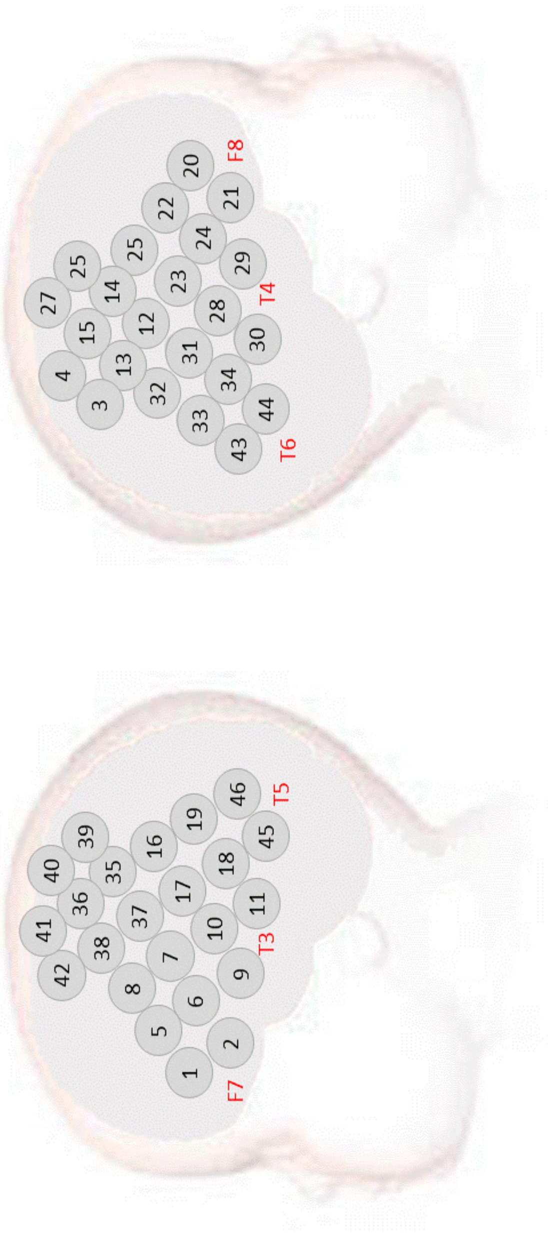
F8
T4
T6
20 21 22 24 25 29 23 28 27 25 14 15 12 13 31 30 4 3 32 34 33 44 43
T5
F7
T3
46 19 45 39 16 18 40 35 17 11 36 37 10 41 38 7 9 42 8 6 5 2 1

A.

B.

Monolinguals - Spoken language

Left hemisphere

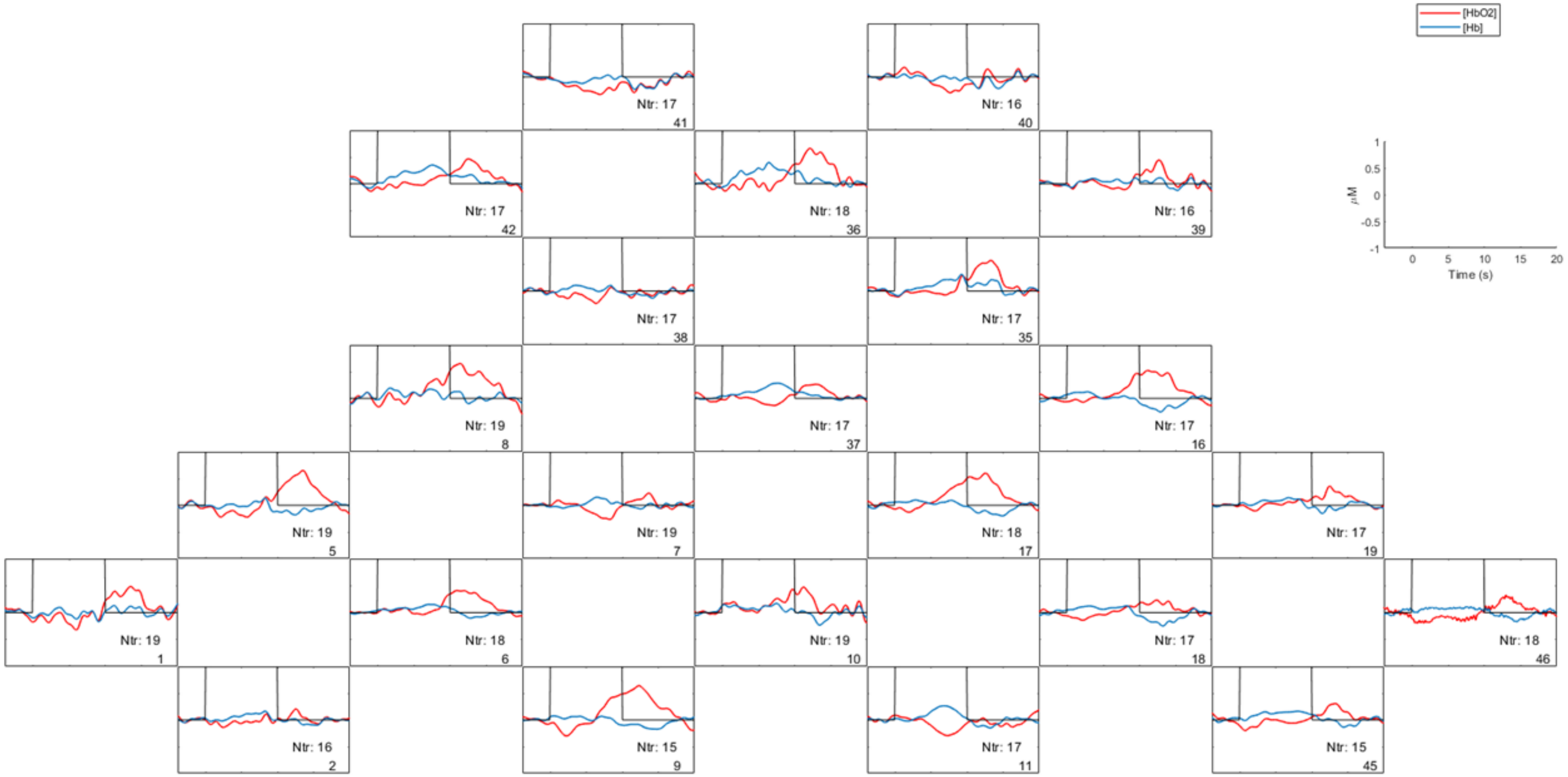

C.

Monolinguals - Spoken language

Right hemisphere

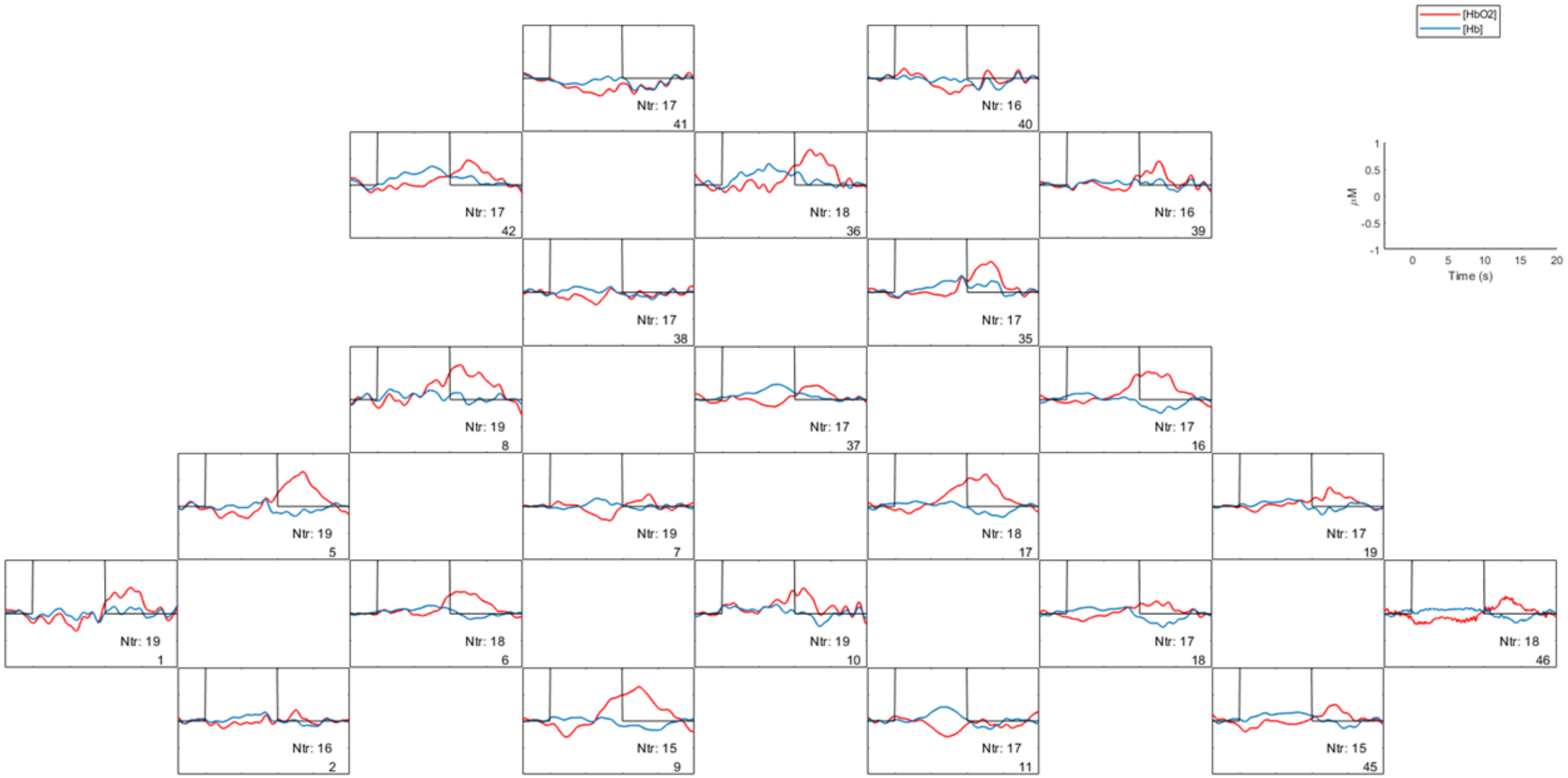

D.

Unimodal bilinguals - Spoken language

Left hemisphere

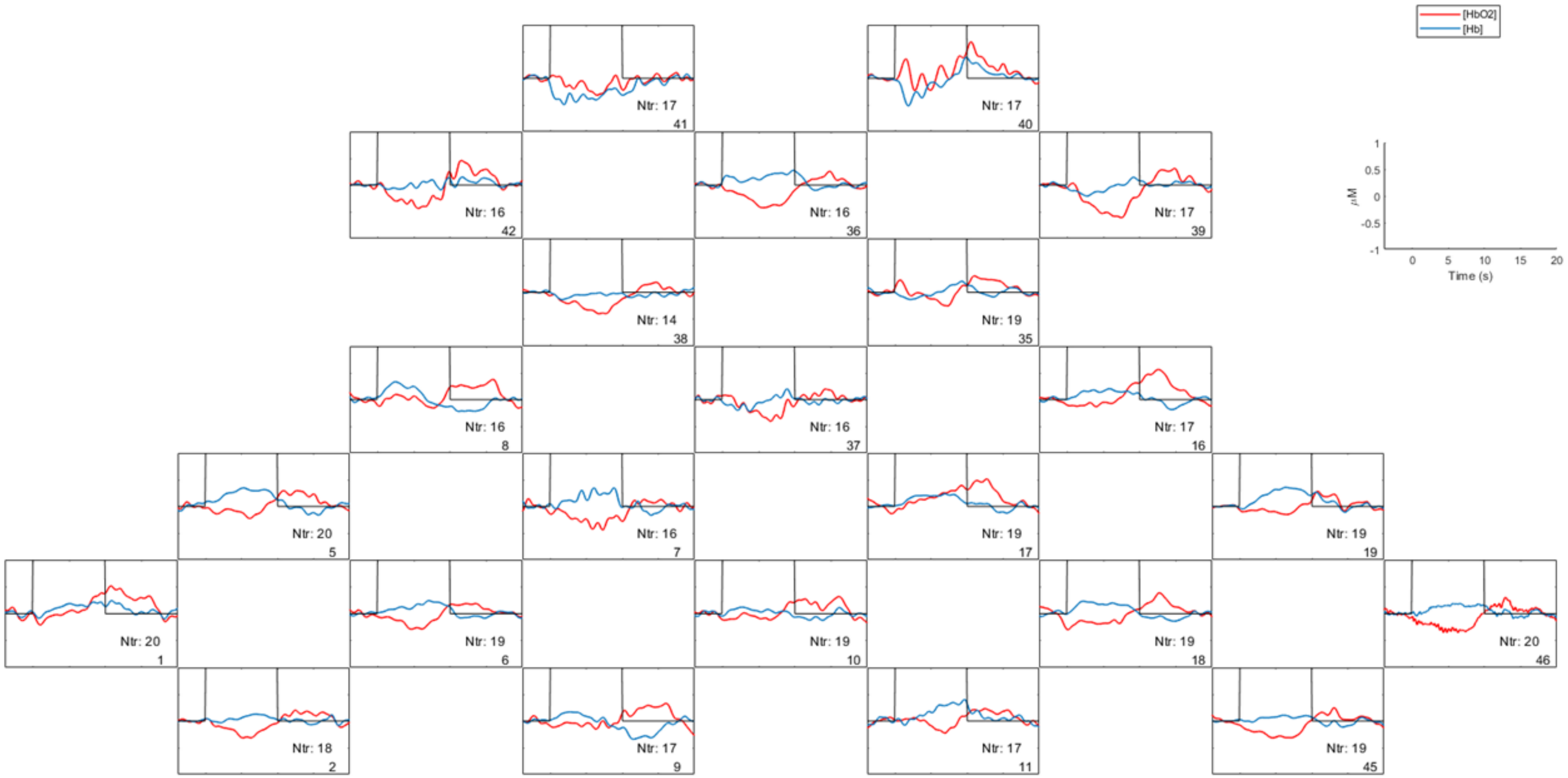

E.

Unimodal bilinguals - Spoken language

Right hemisphere

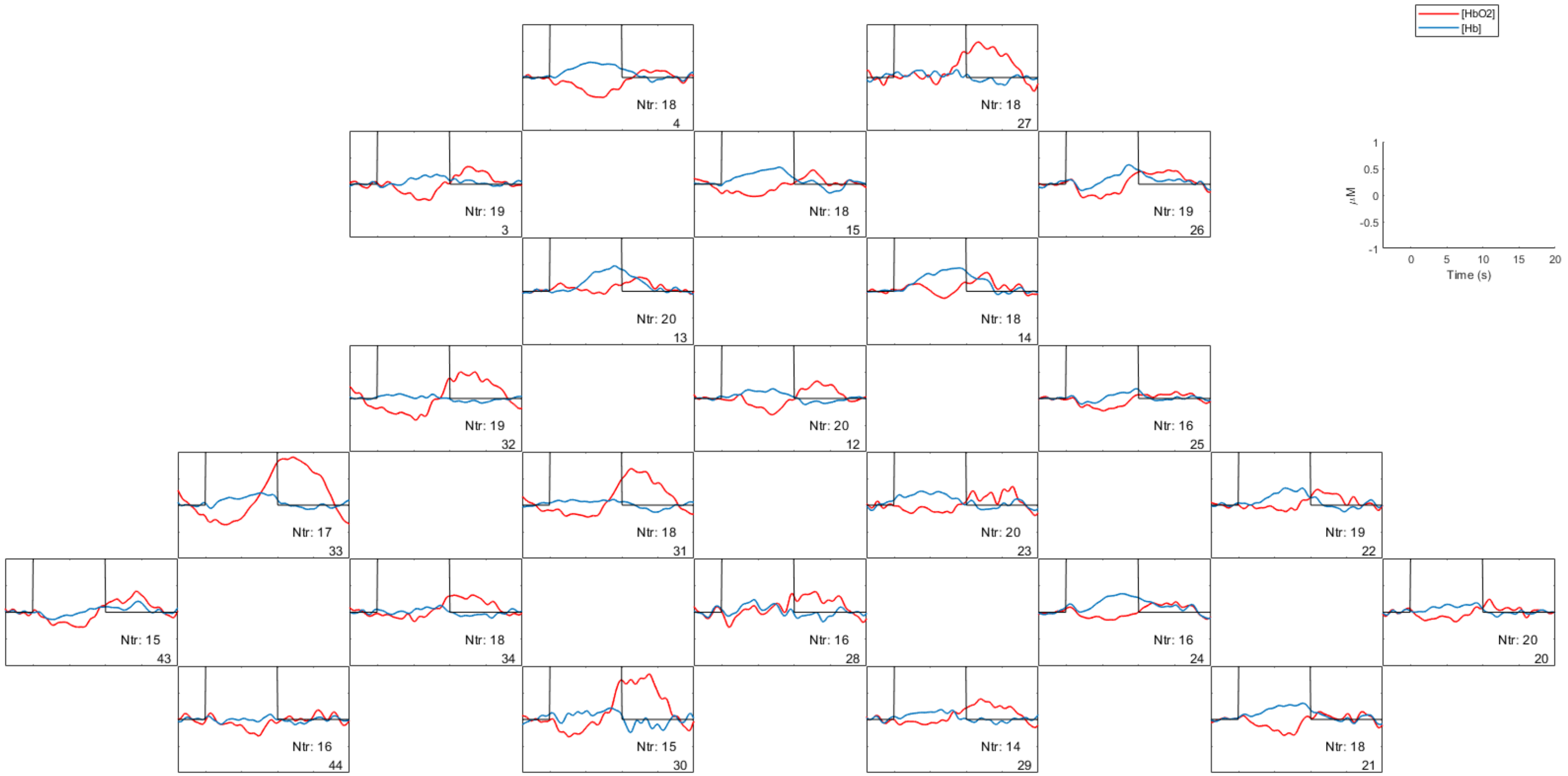

F.

Bimodal bilinguals - Spoken language

Left hemisphere

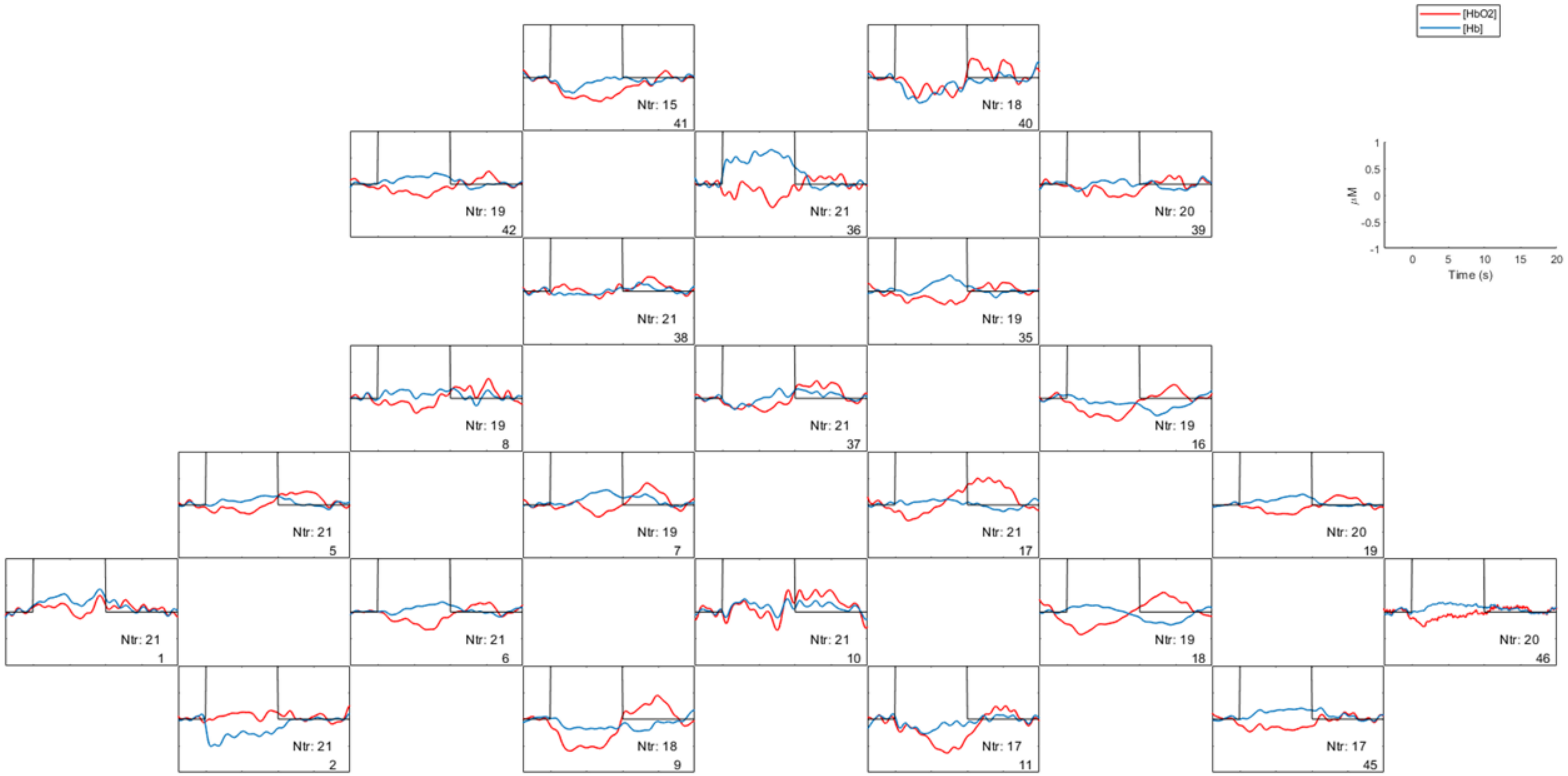

G.

Bimodal bilinguals - Spoken language

Right hemisphere

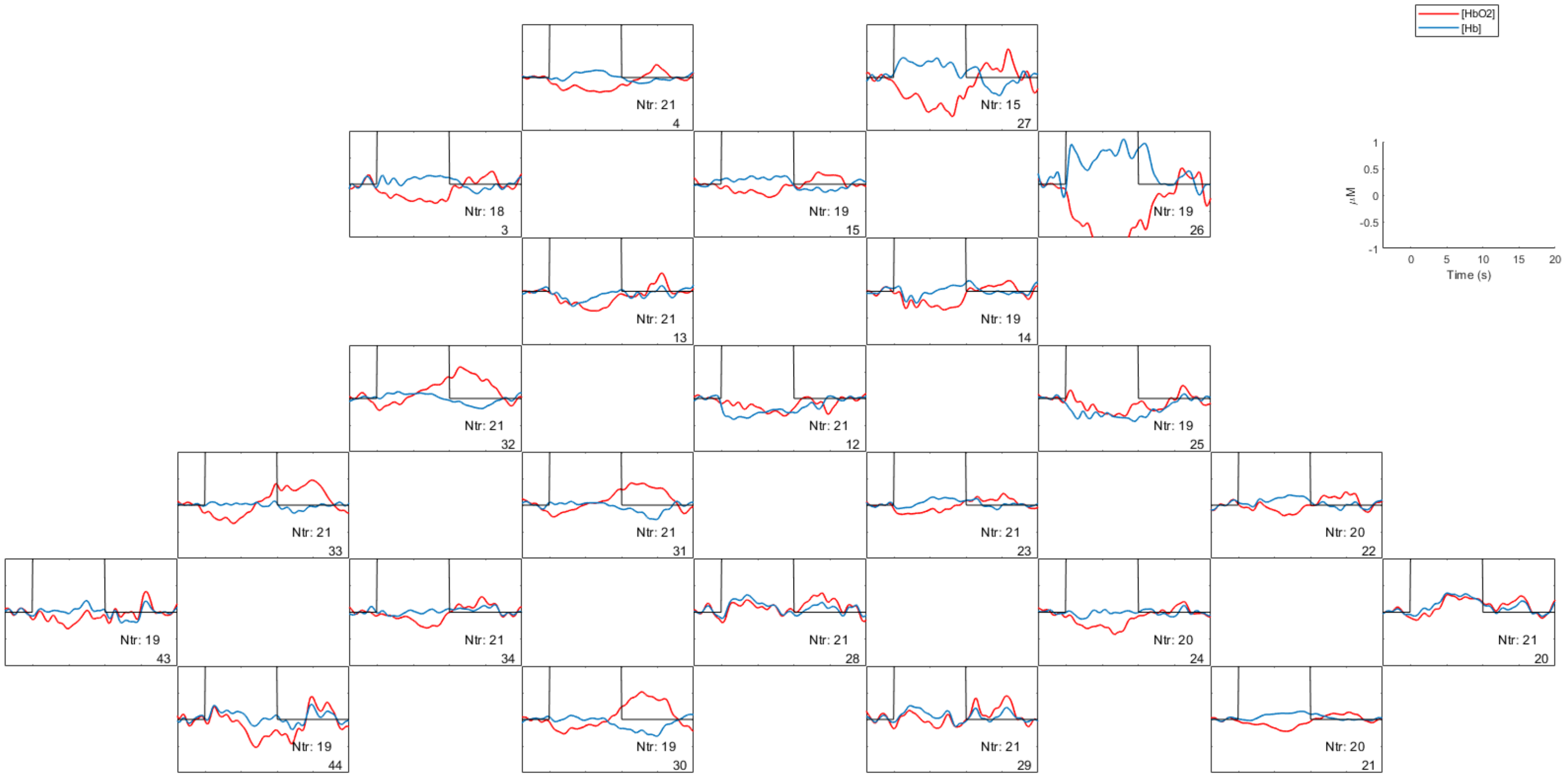

H.

Monolinguals - Signed language

Left hemisphere

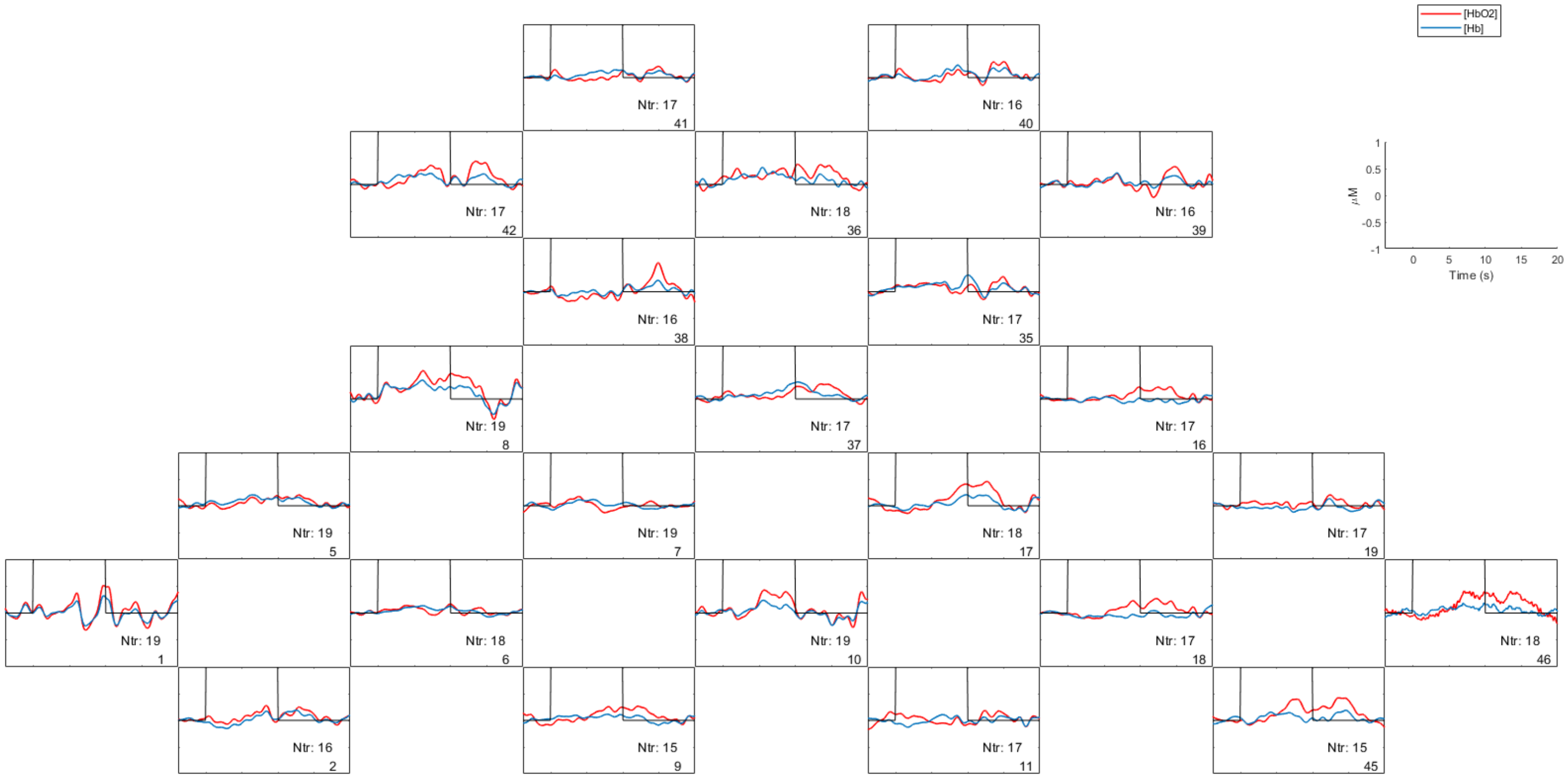

I.

Monolinguals - Signed language

Right hemisphere

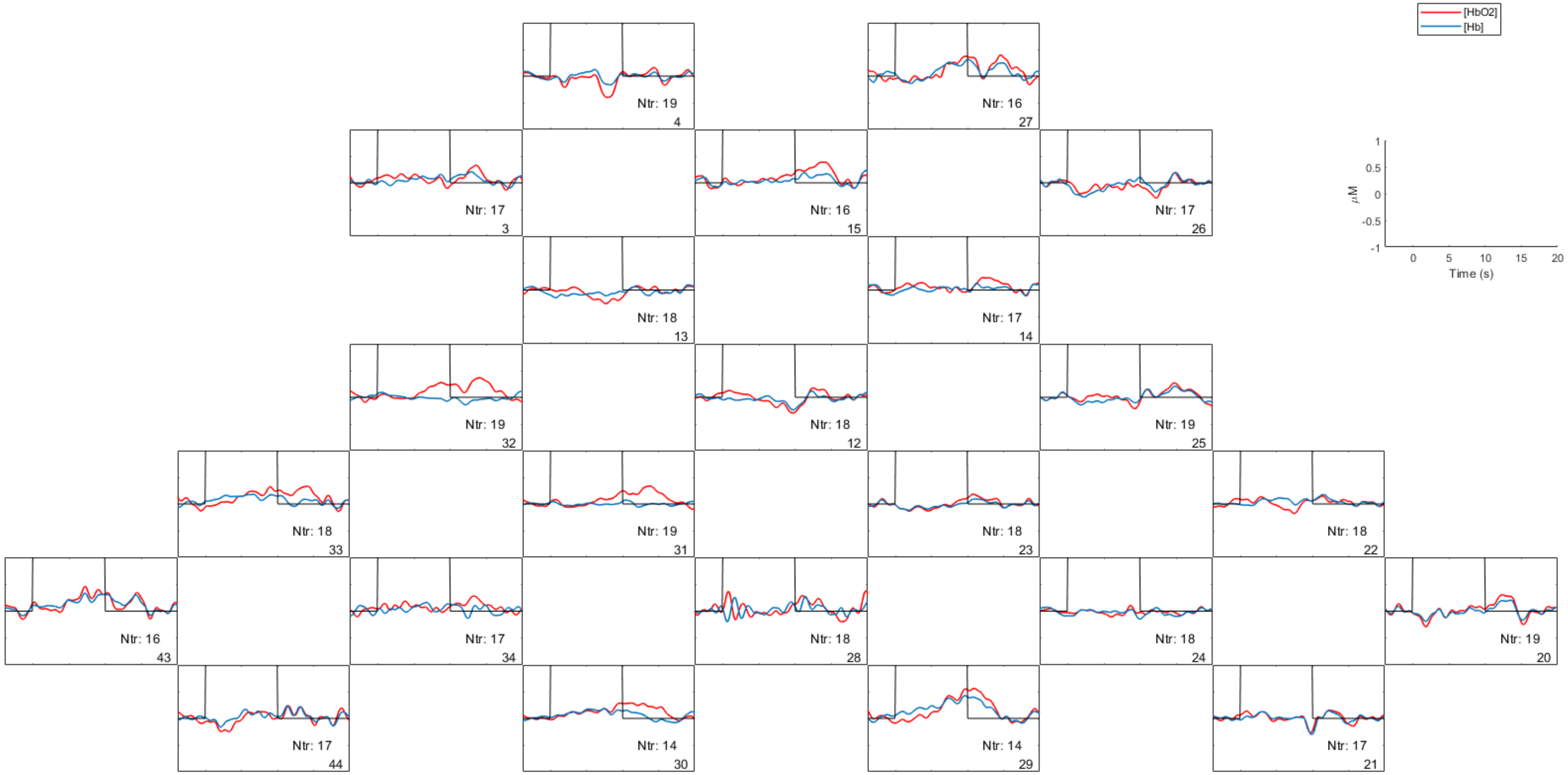

J.

Unimodal bilinguals - Signed language

Left hemisphere

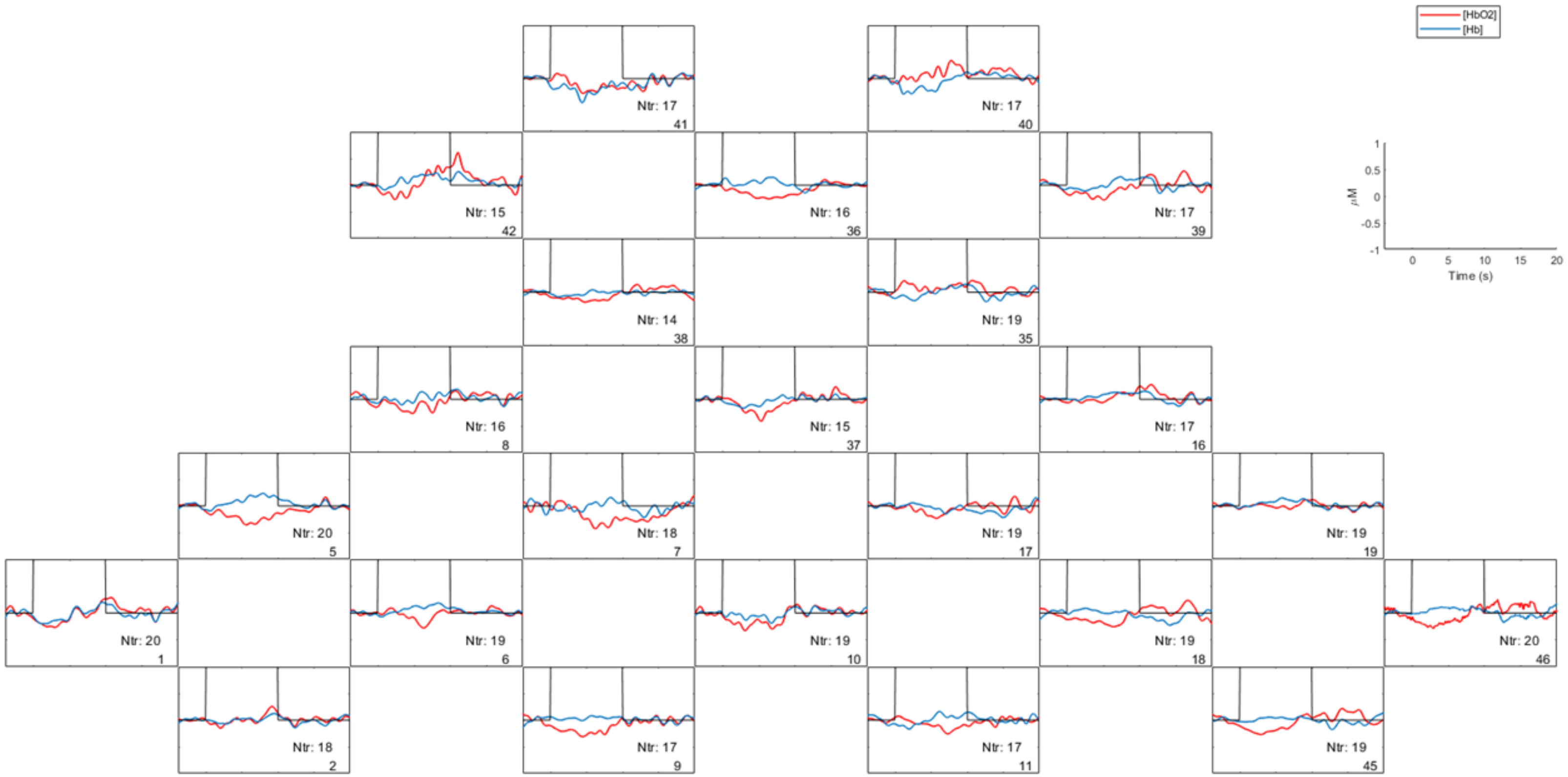

K.

Unimodal bilinguals - Signed language

Right hemisphere

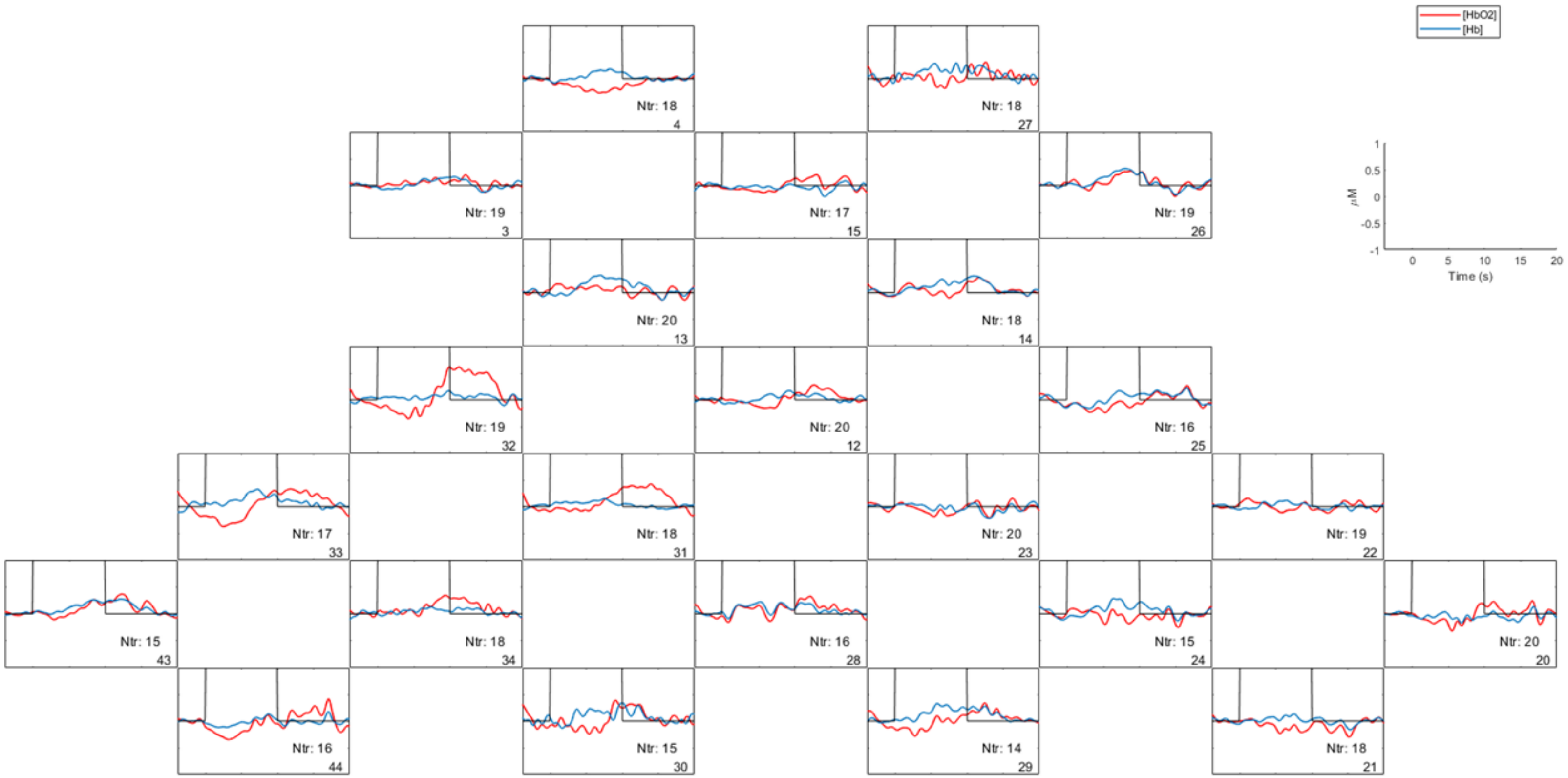

L.

Bimodal bilinguals - Signed language

Left hemisphere

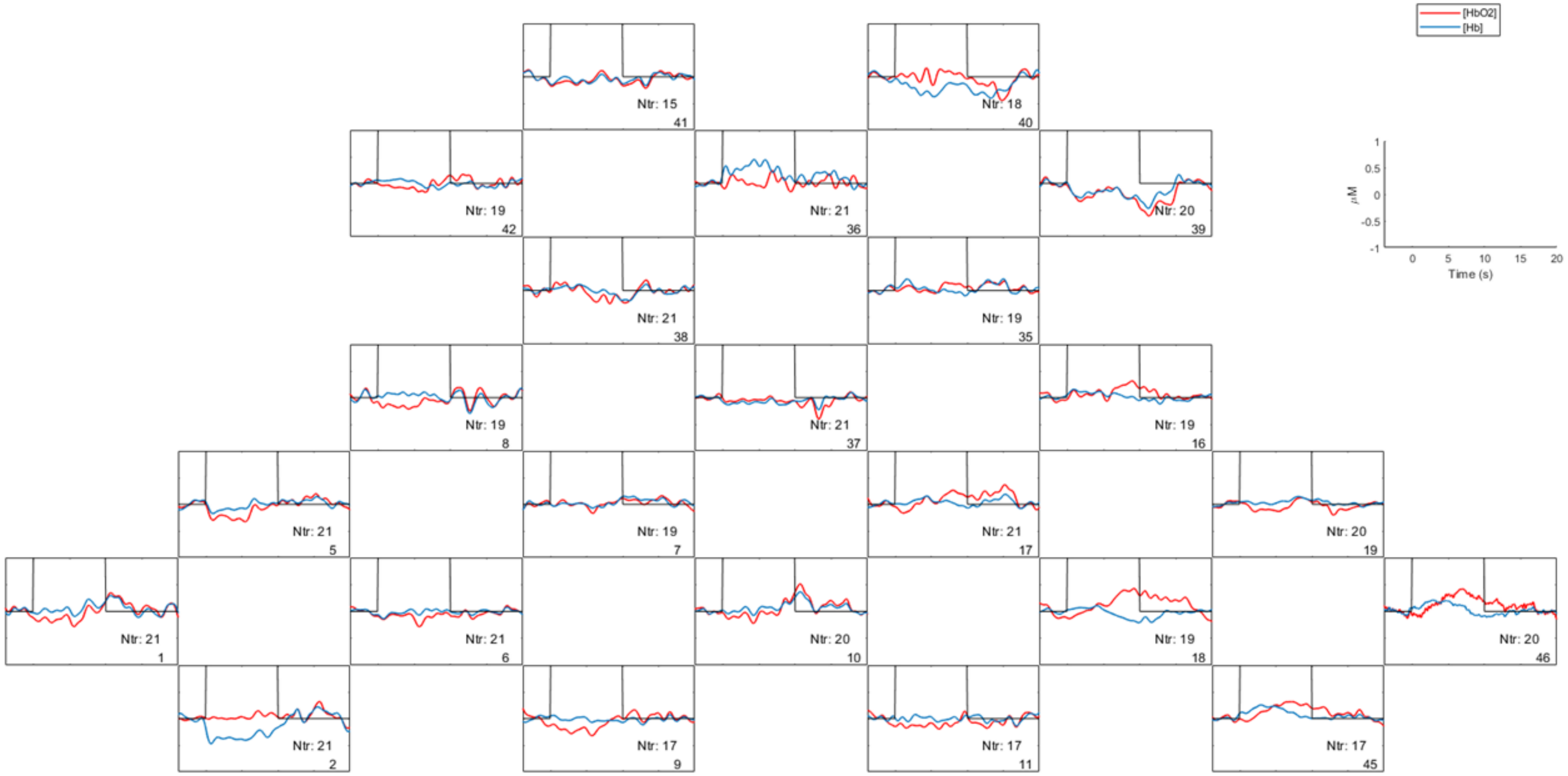

M.

Bimodal bilinguals - Signed language

Right hemisphere

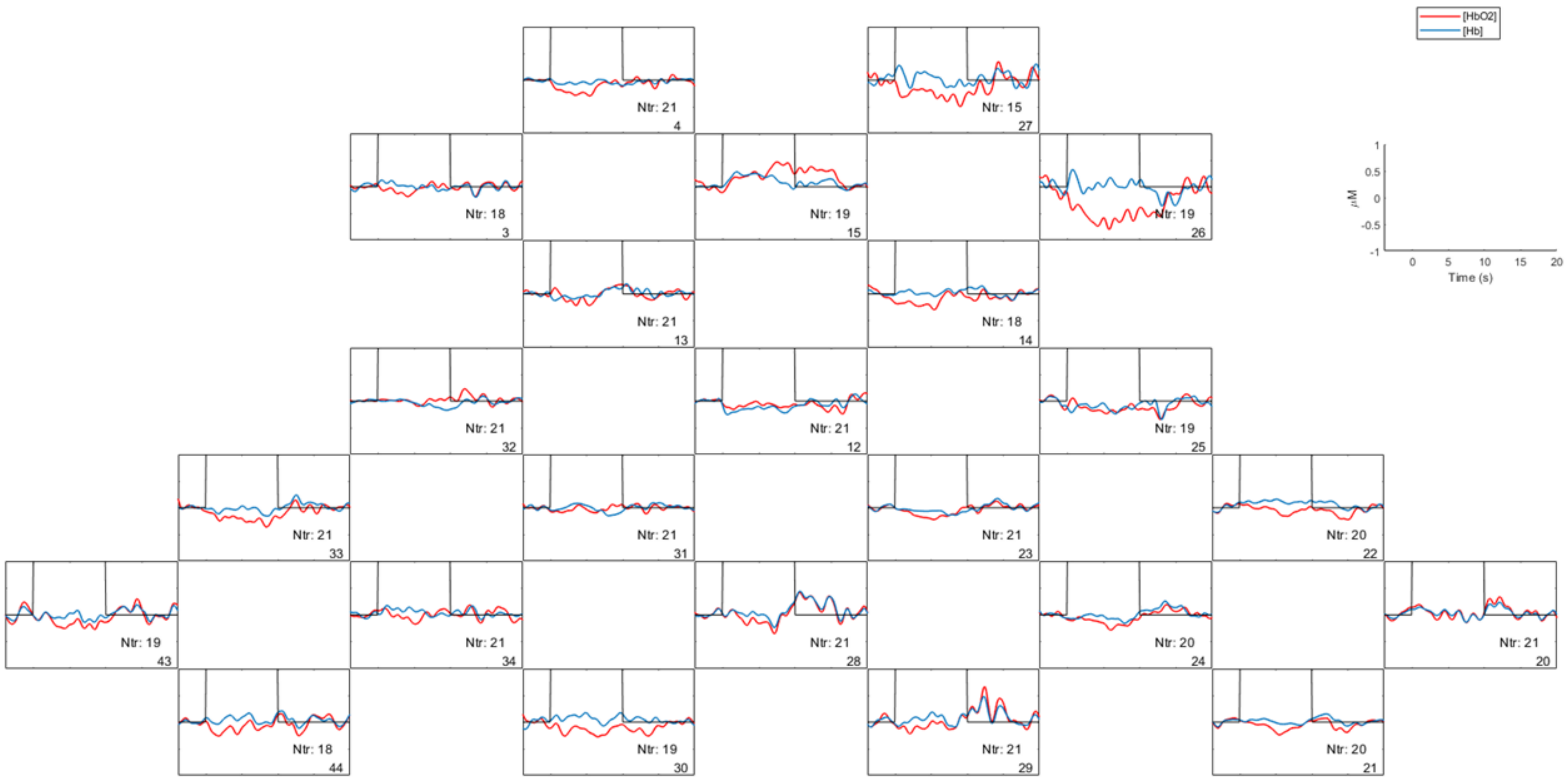

Supplementary Figure 3: Haemodynamic response in each channel and condition. (A) Schema of channel location relative to 10-20 coordinates. (B-M) HbO_2 (in red) and HHb (in blue) grand averaged haemodynamic responses in each group and condition. Ntr = number of included trials.
